# Unfolding molecular switches for salt stress resilience in soybean: recent advances and prospects for salt-tolerant smart plant production

**DOI:** 10.3389/fpls.2023.1162014

**Published:** 2023-04-19

**Authors:** Chen Feng, Hongtao Gao, Yonggang Zhou, Yan Jing, Senquan Li, Zhao Yan, Keheng Xu, Fangxue Zhou, Wenping Zhang, Xinquan Yang, Muhammad Azhar Hussain, Haiyan Li

**Affiliations:** ^1^ College of Life Sciences, Jilin Agricultural University, Changchun, China; ^2^ Hainan Yazhou Bay Seed Laboratory, Sanya Nanfan Research Institute of Hainan University, Sanya, China; ^3^ College of Tropical Crops, Hainan University, Haikou, China; ^4^ School of Chemistry and Chemical Engineering, Guangzhou University, Guangzhou, China

**Keywords:** salt stress, Na +/K + homeostasis, phytohormones, epigenetics, soybean

## Abstract

The increasing sodium salts (NaCl, NaHCO3, NaSO4 etc.) in agricultural soil is a serious global concern for sustainable agricultural production and food security. Soybean is an important food crop, and their cultivation is severely challenged by high salt concentration in soils. Classical transgenic and innovative breeding technologies are immediately needed to engineer salt tolerant soybean plants. Additionally, unfolding the molecular switches and the key components of the soybean salt tolerance network are crucial for soybean salt tolerance improvement. Here we review our understandings of the core salt stress response mechanism in soybean. Recent findings described that salt stress sensing, signalling, ionic homeostasis (Na^+^/K^+^) and osmotic stress adjustment might be important in regulating the soybean salinity stress response. We also evaluated the importance of antiporters and transporters such as Arabidopsis K^+^ Transporter 1 (*AKT1*) potassium channel and the impact of epigenetic modification on soybean salt tolerance. We also review key phytohormones, and osmo-protectants and their role in salt tolerance in soybean. In addition, we discuss the progress of omics technologies for identifying salt stress responsive molecular switches and their targeted engineering for salt tolerance in soybean. This review summarizes recent progress in soybean salt stress functional genomics and way forward for molecular breeding for developing salt-tolerant soybean plant.

## Introduction

Soybean (*Glycine max* (L.) Merr.) is an important legume crop, and over 333 million tonnes are produced globally (Sugiyama, 2019). Soybean, seeds are enriched with essential amino acids, proteins (40%), lipids and metabolites (isoflavones and saponins), contribute 56% of the world’s edible oil for human consumption (Sedivy et al., 2017; Singh et al., 2017). Despite their great importance, field-grown soybean faces great environmental challenges from germination to final harvest. Among these environmental challenges, worldwide soil salinization and alkalinity are growing issues for economic crops, including soybean (Zhao et al., 2020; Feng et al., 2021a). According to current estimations, approximately 1-billion-hectare about 19.5% of farmland is affected by salt worldwide (Li et al., 2019b). In China, more than 100 million hac are under saline-alkaline stress (Cai et al., 2022). In the future, sustainable food supplies will be a serious task to feed a population of 9 billion by 2050 (Zhou et al., 2015). Therefore, breeding soybean varieties with high production, quality and saline-alkaline stress tolerance to guarantee world food security remains an ongoing task.

Soybean is cultivated in tropical, subtropical and temperate climatic regions. The world top ranked soybean producing countries are enlisted in **Table 1**. Soil salinity threatened the soybean’s seed germination, growth and developmental phases. A higher salt concentration causes various damage to soybean, such as high osmotic stress, water loss, homeostasis and ion imbalances. Morphologically, salt-stressed soybean plants exhibited leaf chlorosis, necrosis and scorching (Zhang et al., 2019a). Salt stress affected the nitrogen fixation efficiency by decreasing the number and biomass of root nodules. Soybean yield is significantly affected if the soil salinity exceeds 5 dS/m. Salt stress reduced the quality and quantity of free amino acids, protein, sucrose, and starch content in mature soybean seeds (El-Sabagh et al., 2015; Do et al., 2018). Higher transduction of Na^+^ and Cl^−^ from the root zone caused salt toxicity which decreased upto 40% of soybean yield or complete crop failure (Rasheed et al., 2022). Plants adopted various strategies to minimize the Na^+^ and Cl^−^ ionic/osmotic stress by minimizing the water loss or sequestering toxic ions to storage vacuoles (Deinlein et al., 2014; van Zelm et al., 2020). When Na^+^ enters the main root cell through nonselective cation channels (NSCCs), the salt-mediated signalling starts within 5 min to 5h. Salt-mediated signalling waves of Ca^+^, ROS and 3’,5’-cyclic guanosine monophosphate (cGMP) act as early signalling compounds and make a positive feedback loop for K^+^ influx (van Zelm et al., 2020). Various kinds of ionic transporters/antiporters like Na^+^/H^+^ Exchangers (NHXs), *GmNHX1* and *GmNHX2* have recently been identified that help to regulate the salt concentration in soybean (Sun et al., 2021a). Although various salt sensors and regulators like *GmAKT1* have been reported, additional salt response mechanisms are still required for precise salt-tolerant soybean breeding.

**Table 1:** World top ranked countries for soybean production and cultivation.

Sr. No.	Country	Production (Million metric tons)	Cultivation area (Million hec)
1	Brazil	153.00	41.00
2	USA	116.38	34.94
3	Argentina	45.50	15.9
4	China	20.33	10.27
5	India	12.00	12.70
6	Paraguay	10.00	3.40
7	Canada	6.54	2.37
8	Other	24.26	

Soybean germplasm is highly diverse and natural variations exist for salt tolerance. Recent omics approaches such as transcriptomics, quantitate traits loci (QTL), and genome-wide association studies (GWAS) help to identify various genes that play a significant role in soybean salt stress tolerance (Zhang et al., 2019a). For example, QTLs studies have identified *GmSALT3* and *GmSALT18* genes that have major role in soybean salt tolerance (Guo et al., 2021). These genes could be genetically engineered into soybean germplasm to enhance salt tolerance. For instance, CRISPR/Cas9 mediated editing of *GmAITR*, a negative regulator of salt tolerance, increased the soybean salt stress tolerance (He et al., 2021). Similarly, salt stress affects the epigenetic landscape of gene expression in plants. Recent studies revealed that epigenetic modification, epigenetic memories and AS events are also responsible for genetic variability for salt tolerance in soybean. For example, methylation of GmMYB84 enhanced the soybean salt stress tolerance (Zhang et al., 2020b). Small non-coding RNAs such as miRNA have been identified as critical regulators of salt tolerance through modulation of target gene expression (Islam et al., 2022; Zhang et al., 2022d). For instance, small RNA-Seq analysis of soybean under salt stress treatment identified 17 differentially expressed miRNAs and 31 putative target genes. The miR482bd-5p-HEC1 module was identified as a candidate epigenetic regulatory module in response to salt stress in soybean (Cadavid et al., 2020a). Moreover, miR172c-*Glyma01g39520* module enhanced the water stress and salt stress tolerance by modulating the ABA pathway in soybean (Li et al., 2016). Interestingly, a single miRNA can regulate the expression of multiple genes. Thus, genetic engineering of single miRNA could help to improve multiple traits in soybean. Therefore, the investigation and functional validation of miRNA-target gene module in soybean salt stress should be paid more attention. In response to salt stress, proline, melatonin (N-acetyl-5-methoxytryptamine), glycine betaine (GB) and different sugars had osmo-protective roles and largely accumulated in soybean. For instance, soybean cultivars accumulate more proline content to heal the salt stress damage. However, osmo-protective roles played by proline largely dependent on plant growth and development stages, concentration and duration of salt stress (Mansour and Ali, 2017). Melatonin, a pleiotropic signaling molecule, can relieve salinity stress adverse effects by enhancing soybean germination, growth and development, detoxifying ROS, and regulating stress responsive genes (Wei et al., 2015; Imran et al., 2021). Similarly, GB acts as an osmolyte and osmo-protectant to enhance soybean salt tolerance. For example, exogenous application of GB enhances the antioxidant activities of superoxide dismutase (SOD), ascorbate peroxidase (APX), catalase (CAT) and peroxidase (POD), to enhance the plants stress tolerance (Hernandez-Leon and Valenzuela-Soto, 2022). Similarly, phytohormones, like methylglyoxal (MG) and reactive oxygen species (ROS) detoxifying ascorbate-glutathione (AsA-GSH) pathway, Brassinosteroide (BR), Gibberellin (GA), Jasmonic acid (JA), and Salicylic acid (SA), which has a cross-talk with master stress regulator ABA, contribute soybean salt stress tolerance (Hossain et al., 2021; Waadt et al., 2022). In future, comprehensive investigations are required to establish the exact role of osmoprotectants, phytohormones and the apprehensive mechanisms by which they enhance soybean salt tolerance.

Therefore, this review covered physiological, molecular and biochemical salt-induced changes from salt perception to downstream signalling at the cellular level in soybean. Subsequently, we highlighted the role of different osmo-protectants and phytohormones in ameliorating the salt effects in soybean. We also explained the natural variation contributing to soybean salt tolerance variability. Finally, we explained the shortcomings and prospects of applying these technologies for developing soybean salt tolerance. Salt tolerance is a complex trait. Therefore, it is imperative to augment different salt stress responsive mechanisms to improve soybean performance under salt adversity.

## The impact of salinity stress on soybean

**Figure 1:**
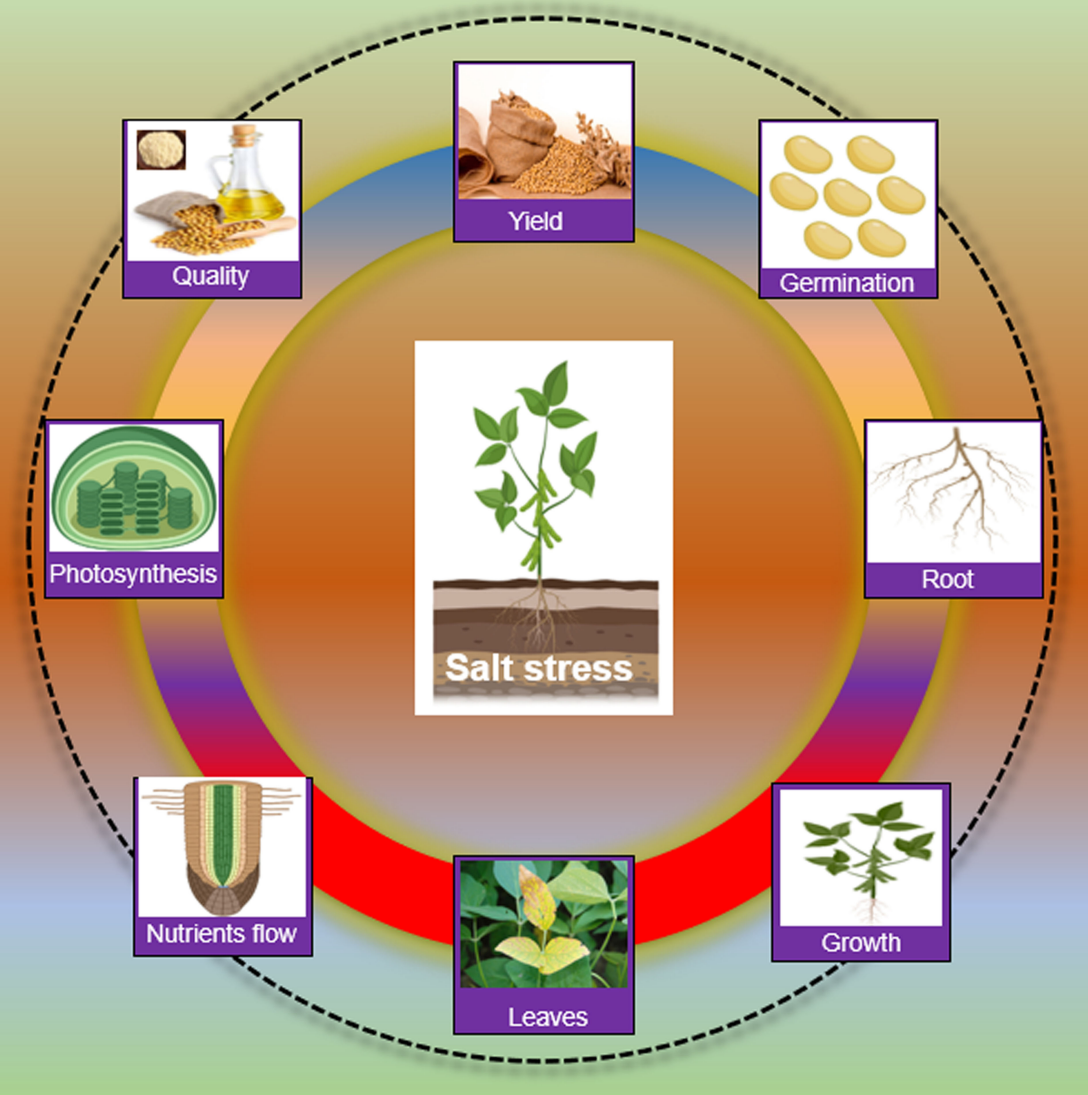
Impact of salt stress on Soybean Salt stress slows down germination, root establishment, seedling growth, causes leaf damage, blockage of nutrient transportation, hinders the photosynthesis process and finally affects the yield and product quality in Soybean.

Salt stress has a broad impact on the morphology and physiology of soybean plants. Salt stress induces ionic stress, particularly (Na^+^/K^+^/Cl^−^ imbalance), osmotic stress (dehydration) and secondary stresses, especially oxidative stress (ROS) (Yang and Guo, 2018). Salt ions caused ionic stress, which is toxic to plant cells. Salts ions move from the root zone to shoots and accumulate in leaves. Higher concentrations of Na^+^ and Cl^−^ ions in leaves imbalanced the cytoplasmic ion levels and metabolic pathways, eventually hindering the photosynthesis process and productivity in soybean (Yang and Guo, 2018; Rasheed et al., 2022; Xu et al., 2022a). Higher salt concentrations in soil or water in the root zone result in hyperosmotic and hyperionic conditions, which reduce the uptake of water or necessary nutrients, thereby causing osmotic stress (van Zelm et al., 2020). Ionic and osmotic stresses lead to secondary stresses such as the accumulation of toxic compounds (ROS) and nutrient imbalances in plant cells under salt stress (Dawood et al., 2022). If not properly managed, generated ROS can be destructive to cellular organs, enzymes, DNA, and lipids. For instance, salt stress disturbs the activity of different enzymes involved in the energy production and conversion process, such as nitrate reductase (NR), glutamate dehydrogenase (GDH) and glutamine synthetase/glutamate synthase (GS/(NADPH) in soybean (Ullah et al., 2019). Phytohormones are critical for plant growth and development and abiotic stress response in plants. Salt stress changed the ABA and GA levels which caused an imbalance in the growth and salt stress response in soybean (Shu et al., 2017; Kataria et al., 2019). Morphologically, salt stress reduced seed germination, seedling emergence, growth, leaf length, plant height, fresh weight, dry biomass, and nodulation of soybean (Shu et al., 2017; Kataria et al., 2019). At the reproduction stage, salt stress affects the pods per plant and the number of seeds per plant in soybean. Salt stress also reduced the quality and quantity of oil and protein contents (**Figure 1**).

## Salt stress perturbs photosynthesis and chloroplast functionality but induced the retrograde signalling in soybean

Photosynthesis is an essential attribute of green plants, which is affected by higher soil salt concentrations. Wild *G. soja* native to saline soil has halophytic nature. Photosystem I (PSI) and PSII (Fv/Fm) photosynthesis efficiency under 300 mM NaCl treatment were significantly decreased in soybean. However, compared to *G. max*, *G. soja* protected the chloroplast ultrastructure and leaf lipid peroxidation by showing rapid recovery after salt stress. Therefore, differences in the photosystems of halophytic soybean could help to understand the salt adaptation mechanisms in cultivated soybean (Yan et al., 2020). Chloroplasts are responsible for photosynthesis related biochemical reactions. Salt stress affects the structures and function of chloroplasts by altering the size, number, lipid and starch accumulation, lamellar organization, and interfering with cross-membrane transduction (Hameed et al., 2021). Salt stress caused an increase in PSI transcripts. PSI is more stress-resistant than PSII. PSI enhanced cyclic electron flow to generate ATP while evading ROS accumulation to impart salt tolerance (Munekage et al., 2004; Johnson, 2011). ATP *via* cyclic electron flow around PSI helped to prevent the transduction of Na^+^ in chloroplasts of soybean (He et al., 2015). Therefore, salt toxicity and inhibition of photosynthesis was linked with the hyperaccumulation of Cl^−^ but not that of Na^+^ in chloroplasts of soybean (Subbarao et al., 2003; Chen and Yu, 2007). Additionally, ROS and chloroplastic metabolites facilitate stress communication signalling between chloroplasts and the nucleus, a process known as ‘retrograde signaling’. Almost 10% to 20% of abiotic stress responsive genes protein are localized to the chloroplasts (Kmiecik et al., 2016). ROS-mediated retrograde signalling depends on factors like generation site, type, dose, timing, and duration in cells. ROS with known retrograde signalling function includes hydrogen peroxide (H_2_O_2_), singlet oxygen (^1^O_2_), and superoxide anion radical (O^2–^) (Laloi et al., 2004; Corpas et al., 2017). Lower levels of ROS activate the stress acclimation process, while higher accumulation causes cell death under stress conditions. For example, in response to stress, generated H_2_O_2_ is transduced into the nucleus through H_2_O_2_-specific aquaporin channels, known as peroxiporins. In the nucleus, H_2_O_2_ targets and activates various TFs and stress responsive genes such as DREBs, HSP, WRKY, and cytochrome P450. H_2_O_2_ caused activation of either quick response and, afterward induction of many downstream target genes for stress response (Crawford et al., 2018).

## Mechanisms of calcium influx and salt stress signalling

**Figure 2:**
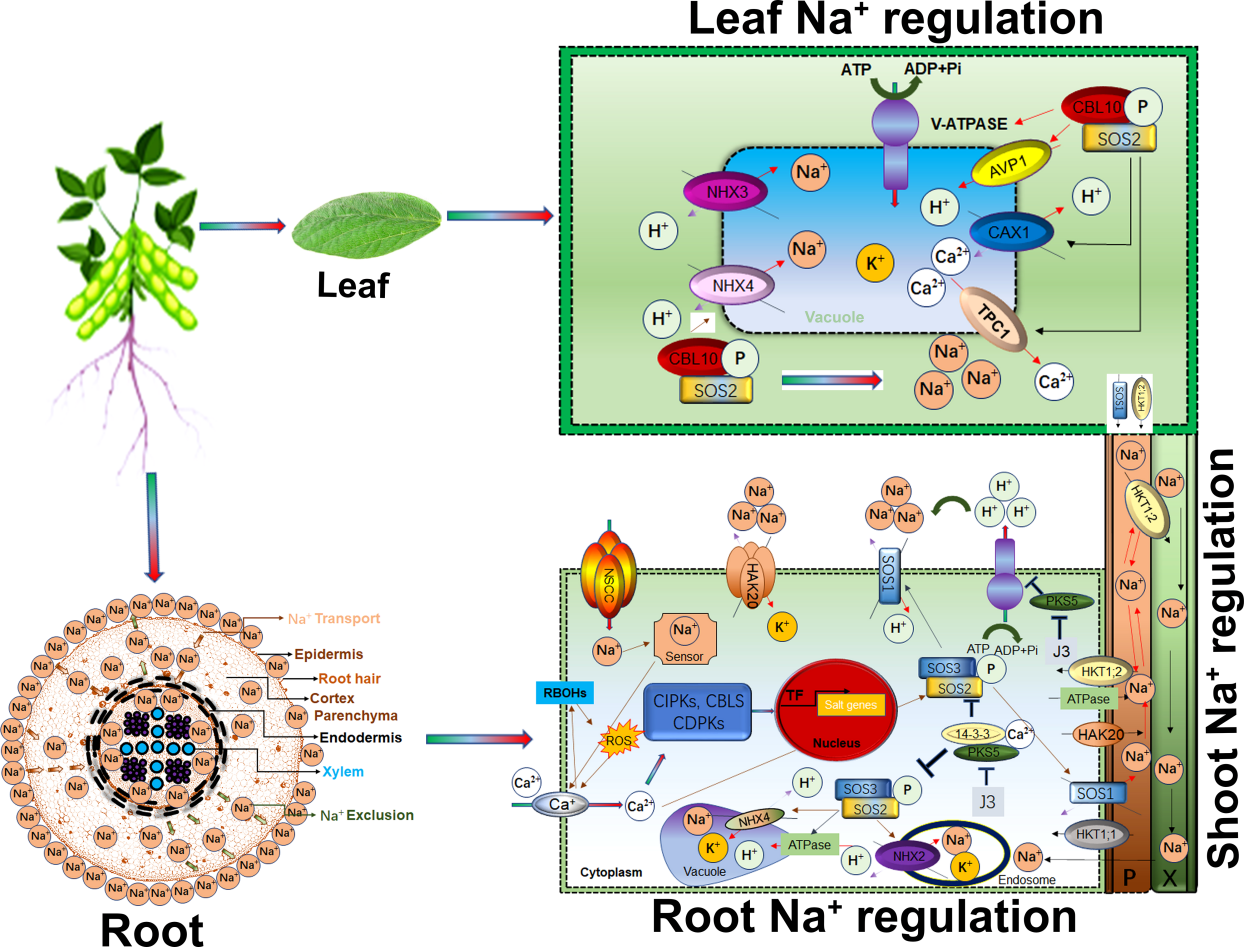
Salt (Na^+^) regulation in root, shoots transportation and leaves response Higher Na^+^ concentration around the root epidermis is eventually transported through membrane pores. Apoplastic and symplastic pathways are critical in Na^+^ ions movement in membrane. Ca^2+^, RBOHs and ROS cascade induces Ca^2+^ import upon higher accumulation of Na^+^ through NSCC within cytoplasm. Ca^2+^ mediates alteration of CIPKs-CBLs-CDPKs modules transcriptional profile thus enhanced the Na^+^/H^+^ and K^+^/H^+^ transportation and vacuole storage. SOS2-SOS3 complex activate the SOS1 for Na^+^ partitioning, exclusion and transportation to leaves. In leaves, CBL10-SOS2 complex trigger the storage or Na^+^ exclusion to shoots by activation of different transporters. HKT1;2 is involved Na^+^ recirculation from shoots to roots.

Calcium (Ca^2+^) is a universal secondary messenger at the cellular level. Ca^2+^ initiates many cellular responses to deal with biotic and abiotic stresses. For instance, salt stress induced early response signalling is mediated by Ca^2+^ waves, cGMP, and ROS to trigger downstream salt tolerance related genetic pathways and for cell expansion to minimize the stress effects. Recently, Na^+^ specific, Ca^2+^ wave influx was identified in plant root cells (Choi et al., 2014). Roots have sodium-specific sensors that regulate root growth direction in saline soil (Galvan-Ampudia et al., 2013). As shown in **Figure 2**, root plasma membrane-associated NSCCs channels transduce Na^+^ in plant cells (Demidchik and Tester, 2002; Demidchik and Maathuis, 2007). Salt-induced Ca^2+^, cGMP and ROS signals regulate NSCCs channel activity. Additionally, plant roots are also inbuilt with extracellular salt sensors such as MONO CATION INDUCED [Ca^2+^] INCREASES 1 (MOCA1) that sense Na^+^ ions (Jiang et al., 2019). MOCA1 generates glycosyl inositol phosphorylceramide (GIPC) sphingolipids at the plasma membrane that open unknown channels for Ca^2+^ influx. The *moca1* mutant cannot produce Ca^2+^ waves in response to Na^+^, K^+^, or Li^+^ ions accumulation and is thus sensitive to salt stress (Kiegle et al., 2000; Donaldson et al., 2004; Miller et al., 2010). Vacuolar Ca^2+^ is influx by TWO-PORE CHANNEL1 (TPC1) for long-range calcium signals (**Figure 2**). The *tpc1* mutants were found inefficient in speed and long-range Ca^2+^ wave signals in response to stress (Choi et al., 2014). CBL mediates CIPKs phosphorylation for Ca^2+^ outfluxes in response to salt stress (Manishankar et al., 2018). Under salt stress, Ca^2+^ signal-activates SOS pathway for sodium ion homeostasis in cells. Calcium-mediated SOS pathway help to exclude Na^+^ from cells. For example, Ca^2+^ mediates *SOS3*/*CBL4* interaction with *SOS2*/*CIPK24*. The *SOS2*-*SOS3* complex regulates the phosphorylation of H^+^/cation antiporter *SOS1*/*NHX7* to release Na^+^ out of cells (Liu and Zhu, 1998; Halfter et al., 2000; Liu et al., 2000). Similarly, the *CBL4*-*SOSO2* complex squeezes Na^+^ in the vacuole. The SOS2-LIKE PROTEIN KINASE5 (*PKS5*) can phosphorylate SOS2. Under salt stress, Ca^2+^ binds the 14–3–3 proteins and represses the PKS5 activity, thus attenuating the SOS2 (**Figure 2**). SOS2 caused the phosphorylation of *AtANN4* to generate a salt-specific calcium signal (Ma et al., 2019). CPK3-mediated phosphorylation of the vacuolar TWO-PORE K^+^ CHANNEL 1 (*TPK1*), or knockout cpk3 and tpk1 mutants, were found salt-sensitive (Latz et al., 2013). A recent study reported that salt-tolerant soybeans strongly activated *CBL10*-*CIPK24*-*NHX* and *CBL4*-*CIPK24*-*SOS1* complexes to reduce salinity effects (He et al., 2015). Exogenous application of gibberellin biosynthesis inhibitor prohexadione-calcium (Pro-Ca) effectively protects saline-alkali stress damage by regulating seedling phenotype, photosynthetic apparatus, antioxidant defense, and osmoregulation (Feng et al., 2021b).

## Maintaining Na^+^/K^+^ ionic homeostasis in response to salt stress in soybean

Cytosolic pH homeostasis is indispensable for plant growth and abiotic stress responses (Zhou et al., 2021). Proton pumps-maintained pH homeostasis through cellular ion transport and Na^+^ sequestration (Li et al., 2022b). P-typeATPases (P-ATPases) are localized in the cellular membrane and improved the Na^+^ sequestration in the vacuole (**Figure 2**). Similarly, P-ATPases mediated enhanced Na^+^/proton antiporter *SOS1*/*NHX7* activity increased the salt and alkaline stresses tolerance through Na^+^ exclusion from root (van Zelm et al., 2020). The cellular homeostasis between Na^+^ and K^+^ is maintained by replacing K^+^ with Na^+,^ which is mediated by ion channels, transporters, and antiporters during salt stress (**Figure 2**). The *GmNHX1* localized on vacuolar membranes, predominantly transports Na^+^ to leaves and reduces Na^+^ absorption in roots. Soybean plants overexpressing or knockout *GmNHX1* exhibited higher salt tolerance or susceptibility, respectively. Additionally, *GmNHX1* transformed plants showed higher Na^+^ efflux rate and maintained a higher K^+^/Na^+^ ratio after salt treatment than wild-type in addition to induction of osmotic stress-related genes, SKOR, SOS1 and *AKT1* to enhance soybean salt tolerance (Sun et al., 2019b). Apart from the vacuolar membranes localized *GmNHX1*, the other members of the NHX family *GmNHX5* localized to Golgi apparatus can transport both K^+^ and Na^+^, maintaining a higher Na^+^/K^+^ ratio which has demonstrated relevance for salt tolerance in soybean. Mechanistically, *GmNHX5* also regulates osmotic stress-related genes, such as *GmSOS1*, *GmSKOR*, and *GmHKT1* by maintaining a higher Na^+^/K^+^ ratio to improve the salt tolerance compared to CRISPR/Cas9 mediated knockout *GmNHX5* Soybean (Sun et al., 2021a). The promoter of *GmNHXs* contained Amiloride binding motifs, an inhibitor of Na^+^/H^+^ exchange activity in soybean. Induction of *GmNHX2* after 200 mM NaCl stress also indicated its significant role in salt tolerance, which needs to be determined in soybean (Joshi et al., 2021). *GmSOS1* localized in the plasma membrane is a Na^+^ efflux transporter in salt stress. CRISPR-Cas9 mediated *gmsos1* mutants that significantly accumulated higher Na^+^ in the roots resulting in the imbalance of Na^+^ and K^+^ efflux under salt stress (**Figure 2**). *GmSOS1* is a Na^+^/H^+^ transporter and plays a critical role in soybean salt tolerance by maintaining Na^+^ homeostasis (Zhang et al., 2022a). Further, the Cation/H^+^ -exchanger (CHX) had a protective role in salt stress. In soybean a major QTL for salt-tolerance has the causal gene *GmCHX1* which is a close paralog of *GmCHX20a*. Interestingly, overexpression of *GmCHX20a* led to increased salt sensitivity mainly due to increasing Na^+^ uptake into the root. However, on the contrary, *GmCHX1* overexpression enhanced salt tolerance *via* Na^+^ exclusion under salt stress. Thus, the concerted effects of *GmCHX20a* and *GmCHX1* reduced the osmotic and ionic stress in response to elevated salinity in soybean (Jia et al., 2021). However, our current knowledge is limited to transporters that have a role in Na^+^/K^+^ homeostasis. In response to salt stress, several cation transporters change their expression; however, their biological role is still elusive in soybean. Recently the development of high-resolution molecular reporters provided deep insights into calcium transport and *in vivo* function. Similar high-resolution molecular reporters for Na^+^ and K^+^ could yield insights into the functions of transporters and the relevance of tissue-specific Na^+^/K^+^ regulation and function (van Zelm et al., 2020).

## AKTI as a master regulator of salt tolerance

**Figure 3:**
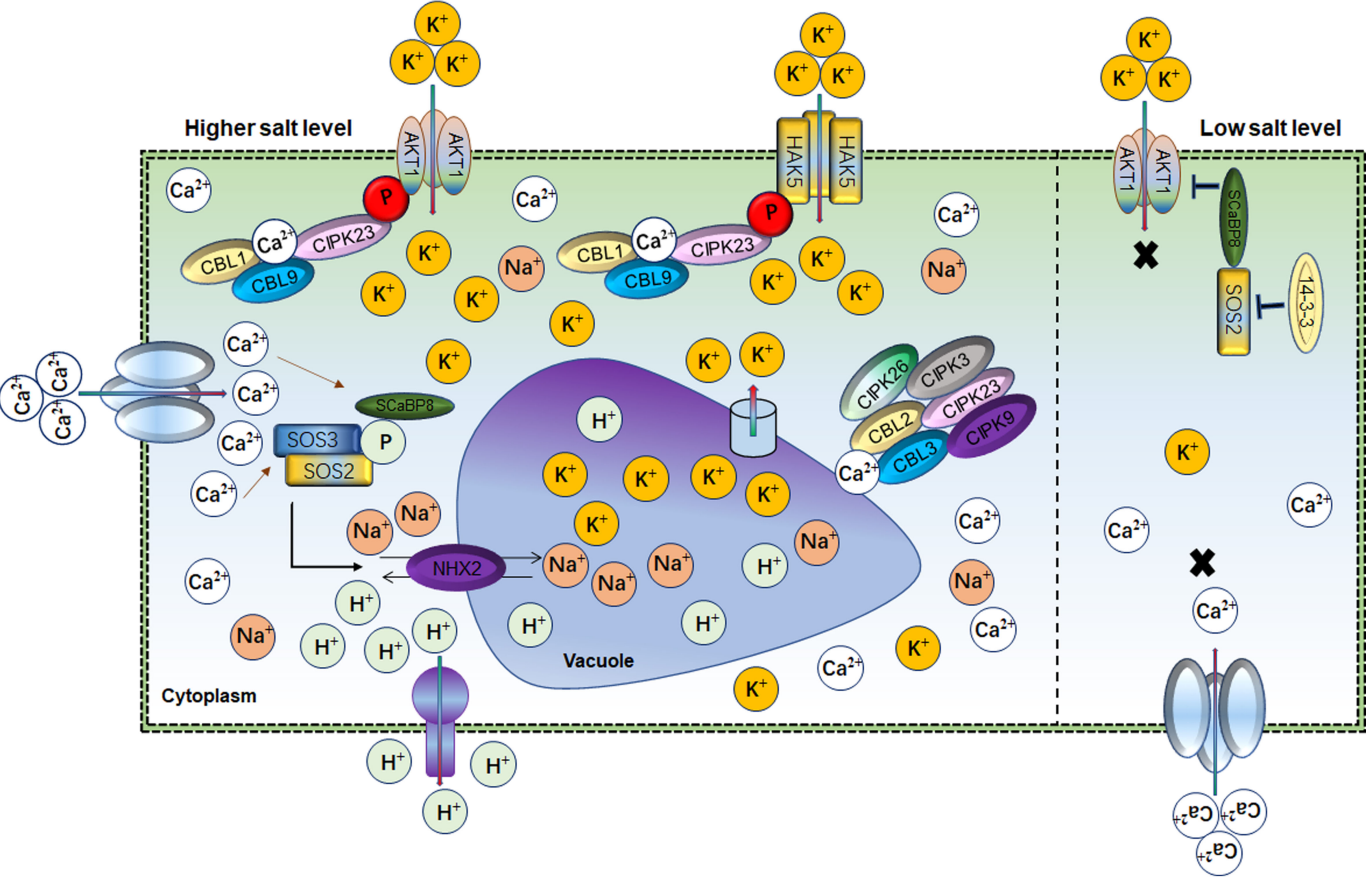
Role of Ca^2+^ and AKT1 channels in balancing the Na^+^/K^+^ homeostasis Cellular ionic homeostasis under lower and higher salt conditions mediated by Ca^2+^ and AKT1 channels. 14-3-3-SOS2- SCaBP8 module blocked AKT1 activity under low salt levels thus inhibiting the K^+^ import into the cell. However, under salt stress, Ca^2+^ activates SOS2 for SCaBP8 phosphorylation to separate from AKT1. SOS2 also had a key role in Na^+^/H^+^ vacuolar partitioning. At the same time, the CBL–CIPK cascade activated across the differential gradients of K^+^, which mediate Ca^2+^ influx through AKT1 and HAK5 to increase cellular K^+^. In parallel, vacuolar K^+^ remobilized through the interaction of CBL2/3 with four CIPKs through K^+^ channels to maintain the K^+^/Na^+^ homeostasis.

Potassium (K^+^) is an essential macronutrient involved in plant drought and salt response (Xu et al., 2006; Wang and Wu, 2013). Physiologically and genetically K^+^ regulation occurs through K^+^ channels (Shaker K^+^) and K^+^ transporters under abiotic stress in plants (Véry and Sentenac, 2003; Véry et al., 2014). In saline soil, plant survival depends on the cellular balance between Na^+^ and K^+^ ratio. The intra-cellular K^+^ level is regulated through high- and low-affinity systems depending upon the external concentration of K^+^ (Voelker et al., 2010). Although molecular similarity exists between K^+^ and Na^+^, they have different roles in cellular processes responding to salt stress. In plants, excessive Na^+^ accumulation causes K^+^ deficiency under salt stress (Assaha et al., 2017). Therefore, maintaining a high K^+^/Na^+^ ratio is a permissive task for plants to survive under salt-stress conditions. A hyperpolarization-activated voltage-dependent K^+^ channel known as *AKT1* maintains the intracellular Na^+^/K^+^ ratio under salt stress (Ardie et al., 2010; Assaha et al., 2017; Ma et al., 2017). *AKT1*, the first characterized K^+^ channel in Arabidopsis, is responsible for cellular K^+^ efflux/influx during salt/osmotic stresses (**Figure 3**). Salt stress impairs *AKT1* channel activity and K^+^ uptake in the *sos1* mutant (Qi and Spalding, 2004). Similarly, *SCaBP8/CBL10* inhibits *AKT1* activity under salt stress (Ren et al., 2013). Arabidopsis *akt1* mutant seedlings were found sensitive to salt stress but could be rescued by exogenous K^+^ application (Qi and Spalding, 2004). *AtKC1* also negatively regulates *AKT1*-mediated K^+^ uptake in Arabidopsis (Wang and Wu, 2013). *AKT1* mutant showed an imbalance K^+^/Na^+^ ratio and salt sensitivity in *Zygophyllum xanthoxylum* (Ma et al., 2017). However, complementation of Arabidopsis *akt1* mutant with *SmAKT1* recovered the salt-tolerant phenotype *via* enhanced K^+^ uptake (Li et al., 2019b). Overexpression of *GmAKT1*, *OsAKT1* and *HvAKT1* improved the root zone K^+^ uptake and subsequently removed the salt stress effects in soybean, rice and barley, respectively (Ahmad et al., 2016a; Feng et al., 2020b; Feng et al., 2021a; Wang et al., 2021a). Physiologically, intracellular K^+^ concentration determined the *AKT1* phosphorylation state for K^+^ influx under salt conditions. For instance, Calcineurin B-like protein (CBL) mediated cascade such as *CBL1/CBL9* activates CBL-interacting serine/threonine-protein kinase 23 (*CIPK23*) to phosphorylate *AKT1* to boost K^+^ influx in response to lower cellular K^+^ in stress conditions (**Figure 3**) (Xu et al., 2006; Li et al., 2014; Behera et al., 2017). *AKT1* forms either homo or hetero-tetrameric channel with pore-forming subunits or K^+^ channel α-subunit *AtKC1*, respectively. *AtKC1* formed the *AKT1-AtKC1* complex to inhibit the K^+^ transduction activity of *AKT1* which is also dependent on the *CBL1/9-CIPK23-AKT1* phosphorylation cascade (Duby et al., 2008; Wang et al., 2010; Jeanguenin et al., 2011; Wang et al., 2016; Lu et al., 2022). Additionally, various other K^+^ channels-related genes were identified in different plants such as *AKT5*, *SPIK*, *KAT1*, *AKT2*, *KC, SKOR GORK, VvK1.2* and *FaAKT1* (Anderson et al., 1992; Sentenac et al., 1992; Gambale and Uozumi, 2006; Cuéllar et al., 2013; Garriga et al., 2017). However, these Shaker K^+^ channel genes have an independent role; for instance, *AKT1* and *SKOR* are expressed in roots and stem and transduce K^+^ (Lagarde et al., 1996; Johansson et al., 2006). While *GORK* is expressed in guard cells to regulate stomata and *SPIK* is mainly expressed in pollen for K^+^ uptake (Hosy et al., 2003). *OsKAT1* acts in concert with K^+^ channels genes to actively stabilize the cytosolic cation homeostasis, thus protecting cells from salt effects (Obata et al., 2007). These findings suggested that *AKT1* is a master regulator of K^+^ uptake under low K^+^ concentration and maintains the Na^+^/K^+^ homeostasis to ameliorate the salt stress effects (Feng et al., 2021a).

## Sustaining ionic and ROS homeostasis under saline-alkaline stress in soybean

**Figure 4:**
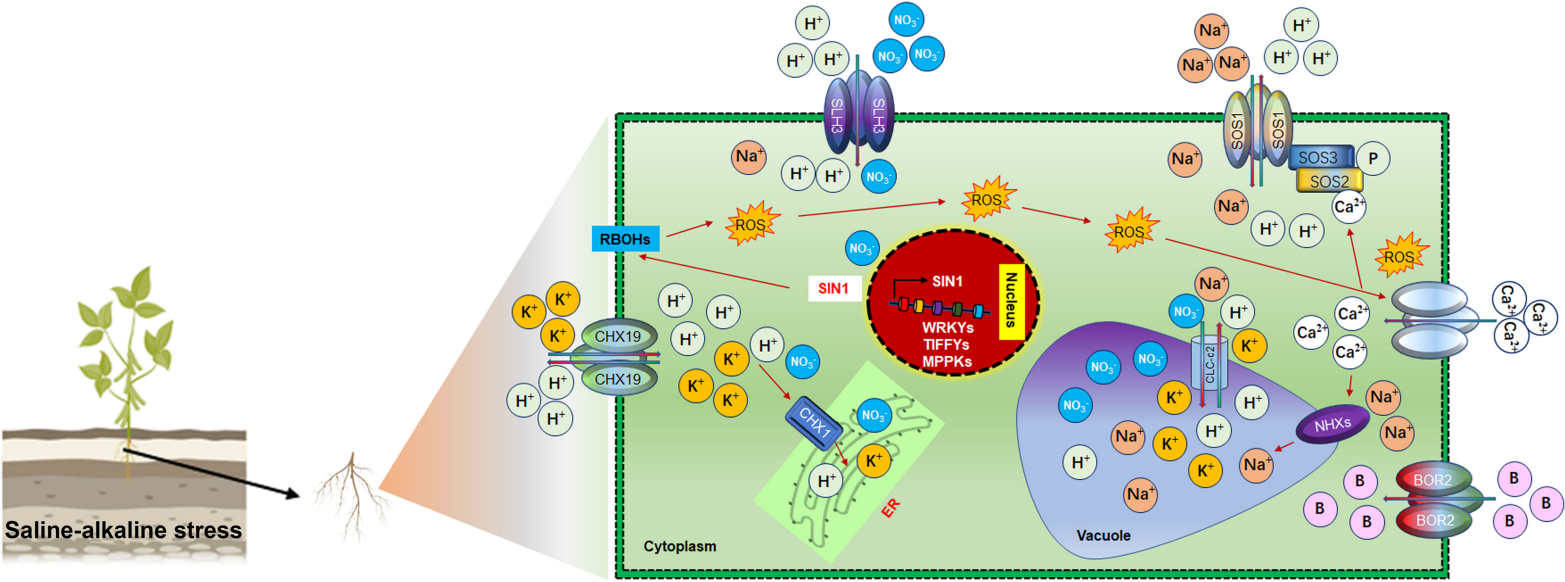
Ionic homeostasis regulation under saline-alkaline stress in Soybean Under saline-alkaline stress, different antiporters and cation exchangers are activated for ionic homeostasis. These transporters transduce ions by choice. For instance, SLH3 is responsible for H^+^ and NO_3_^-^, SOS1 regulate the bidirectional movement of Na^+^ and H^+^ ions, CHX19.3 transduce bidirectional movement of K^+^ and H^+^ into the endoplasmic reticulum (ER) for packaging and subsequently dispose of through budding process form cell. Activation of RBOH by the nuclear SIN1 gene caused the production of ROS. Stimulating the production of ROS under saline-alkaline stress leads to the activation of Ca^2+^ channels to mediate the transduction of ions to the vacuole for storage. Interestingly, most of these regulatory pathways are involved in ionic homeostasis rather than pH regulation under saline-alkaline stress in Soybean.

Investigating the molecular and genetic basis of soybean saline-alkaline stress tolerance is crucial to expand the cultivation and production in saline-alkaline-affected soil. In contrast to salt (NaCl) stress, alkaline stress is exerted by higher soil carbonate (CO_3_^−2^), bicarbonate (NaHCO_3_) and pH, which has more deteriorating effects on soybean growth (Cao et al., 2017). Soil saline-alkaline stress caused ionic imbalance by inhibiting the absorption of K^+^, Ca^2+,^ and Mg^2+^, thus enhancing the Na^+^ and Cl^−^ uptake under higher root zone pH (Guo et al., 2019). Under saline-alkaline stress, root plasma membrane H^+^ deficiency reduces the rhizosphere Na^+^/H^+^ exchange activity, thereby increasing *in vivo* Na^+^ uptake to a hazardous level (Wang et al., 2015a). In planta, saline-alkaline stress induces ionic and osmotic adjustment signals, including Ca^2+^ signals at the cellular level, to reduce the stress effects (**Figure 4**). Ca^2+^ spiking facilitates Na^+^/H^+^ antiporter *SOS1* phosphorylation by the *SOS3-SOS2* complex under sodic stress (El Mahi et al., 2019). Ca^2+^ responsive *SOS1* and plasma membrane H^+^-ATPase modulate Na^+^/H^+^ influx from NaHCO3 to enhance the saline-alkali tolerance (Ni et al., 2020). *SOS2* also triggers tonoplast Na^+^/H^+^ antiporter *NHX1* to squeeze the Na^+^ into the cell vacuole (Assaha et al., 2017). Furthermore, members of the NHX gene, *NHX1-5*, including *GmSOS1*, regulate the compartmentalization of cytoplasmic Na^+^ and K^+^ (Fukuda et al., 2011). *SOS3-SOS2* complex inhibited the *AtHKT1* activity, whereas *SOS4* regulates ion channels and transporters to regulate Na^+^ and K^+^ homeostasis (Shi and Zhu, 2002; Horie et al., 2006). Similarly, plasma membrane localized *GsCHX19.3* regulates the K^+^ and Na^+^ under NaHCO3 stress in soybean (Jia et al., 2017a). In soybean, some genetic factors also regulate NaHCO3 rather than pH. For instance, an alkaline stress-induced slow anion channels homolog (SLAH) *GsSLAH3* and B transporters *GsBOR2* exerted higher tolerance to NaHCO3 and KHCO3 but not to pH (Duan et al., 2018a; Duan et al., 2018b). However, *GsBOR2* direct role in K^+^ regulation under NaHCO3 and KHCO3 stress remained unclear. The *Gshdz4-GsNAC019-GsRD29B* module conferred alkaline stress tolerance rather than high pH (Cao et al., 2016; Cao et al., 2017). Some transporter selectively transduces ion in soybean under saline-alkaline stress (**Figure 4**). For example, *GsCLC-c2* transports Cl^–^ and NO_3_^−^, and *GmCHX1* transport Na^+^, K^+^, and Cl^–^ (Qu et al., 2021) under saline-alkaline conditions (Wu et al., 2018b; Qu et al., 2021). Under alkaline stress, cellular ROS are regulated by modifying the expression of ROS stress signaling genes in soybean. A NAC transcription factor *SALT INDUCED NAC1* (*GmSIN1*) directly induced the expression of *Respiratory burst oxidase homolog B* (*GmRbohB*) to generate ROS to transduce salt stress signals (Li et al., 2019c). Similarly, methionine sulfoxide reductase B (*GsMSRB5a*) could regulate the expression of ROS signaling genes under saline-alkaline stress (Sun et al., 2016). Phytohormones such as Ascorbic acid acts as ROS scavenger under saline-alkaline stress. *L-myoinositol-1-phosphate synthases* (*GsMIPS*) *2* overexpression conferred salt and NaHCO3 stresses tolerance compared to *atmips2* mutant (Chen et al., 2015). The overexpression of *glutathione S-transferases* (*GSTs*) genes, *GsGST13/14/19*, enhanced the saline-alkaline stress tolerance (Wang, 2012; Wang et al., 2012; Jia et al., 2016). In response to salt stress, detoxification of Methylglyoxal (MG) and ROS through MG detoxifying glyoxalase and the ROS detoxifying ascorbate-glutathione (AsA-GSH) pathways played protecting effects against salt stress in plants (Hossain et al., 2021). Glutathione (GSH) is a nonenzymatic antioxidant system activated against abiotic stress in plants. The glutathione peroxidases (GPXs) catalyze the oxidation of GSH to produce oxidized glutathione (GSSG), and convert H_2_O_2_ to H_2_O and alcohol to protect ROS-mediated oxidative damage. In soybean, *GsGPX10.1* and *GsSAMS2* (*S-Adenosyl-L-Methionine Synthetases*) activity is closely related to GSH content and respond to salt stress, indicating a protecting effect by removal of ROS (Hasanuzzaman et al., 2017; Aleem et al., 2022). These studies suggested that GSH content could be increased by overexpressing *SAMS* genes and *GsGPX10.1*, which can help soybean plants to cope with ROS damage. Furthermore, exogenous GSH application minimizes oxidative stress and improves soybean yield-related traits and salt stress tolerance (Akram et al., 2017). Ascorbic acid (AsA) acts as a ROS scavenger in the AsA-GSH cycle. Myo-inositol is one of the main precursors of AsA biosynthesis. MIPSs genes are involved in myo-inositol biosynthesis and respond to NaHCO_3_ and salt stress treatment in soybean. For instance, compared to the GsMIPS2 T-DNA mutant, GsMIPS2 overexpression plants exhibited higher NaHCO_3_ and NaCl stress tolerance in Arabidopsis. These results showed that AsA biosynthesis is critical in salt-alkaline stress tolerance in soybean (Cai et al., 2022). Additionally, different RLKs (receptor-like kinases), SnRKs (sucrose non-fermenting1-related protein kinases), MAPKs (mitogen-activated protein kinases) and transcription factors contributed towards soybean saline and alkaline stresses tolerance. For instance, calcium/calmodulin-binding receptor-like kinase (CBRLK) activated by Ca^2+^/Calmodulins (CaM) complex to form *CBRLK-CPI14* complex to confer the alkaline stress tolerance in soybean (Yang et al., 2010; Sun et al., 2014). Co-overexpression of *GsSnRK1.1* and *GsERF7* significantly improved soybean saline-alkaline stresses tolerance (Feng et al., 2020a). Overexpression of *GsTIFY10*, *GsWRKY20* and *GsWRKY15* also improved the saline-alkaline stress tolerance (Zhu et al., 2011). Although, these genes contribute saline-alkaline stress tolerance in soybean. However, “their exact regulatory mechanisms” such as; How they maintain ionic and ROS homeostasis is still elusive. Identifying their impacts on soybean yields in saline-alkaline fields trials is also imperative.

## Role of osmoprotectants in salt stress tolerance in soybean plants

**Figure 5:**
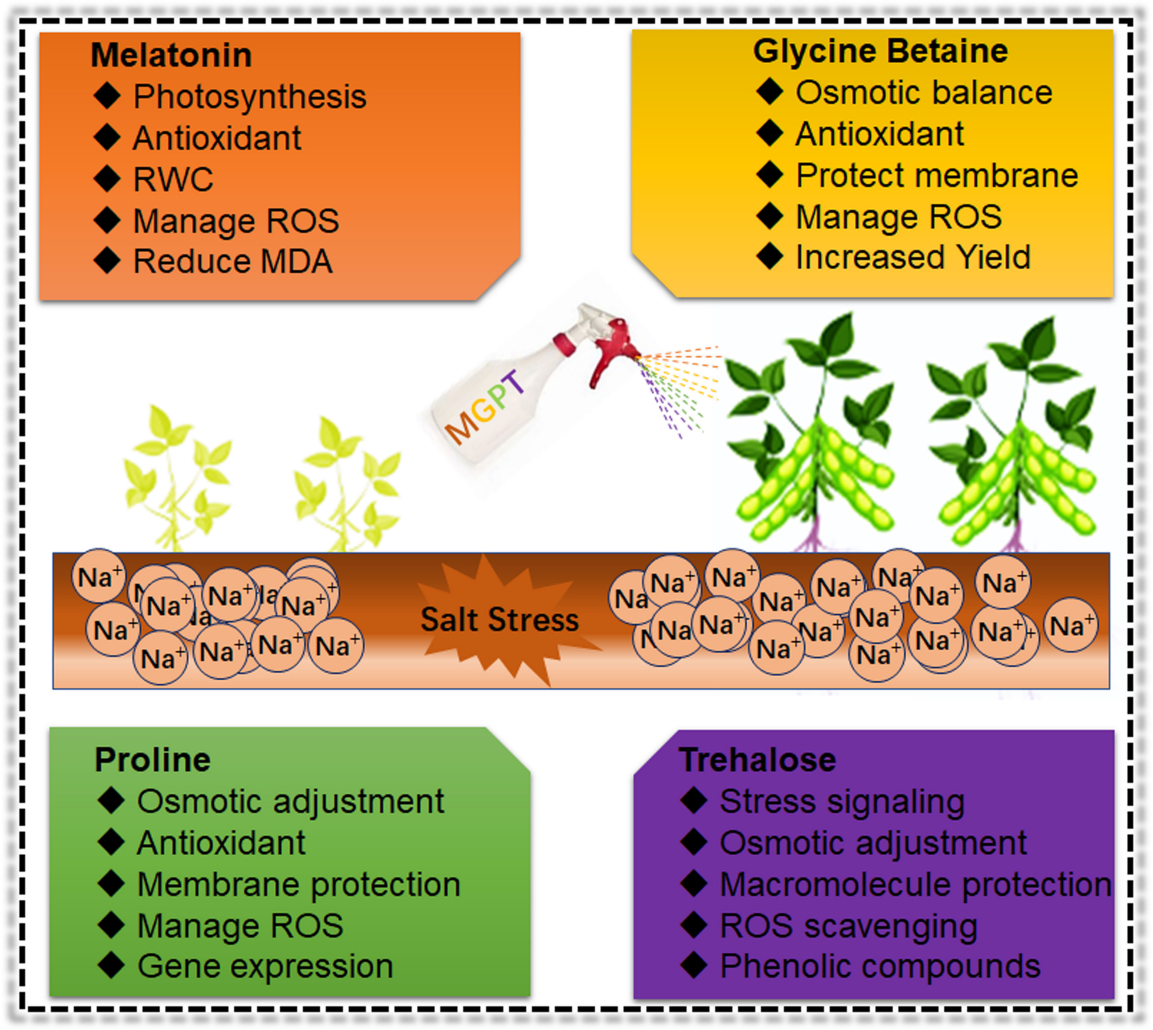
Role of osmoprotectants in ameliorating the Soybean salt stress effects Exogenous application of different biochemical increased the growth and development mainly through enhancement of antioxidant activity, ROS scavenging and protection of cellular structure and membranes under salt stress conditions in Soybean. However, these are short-term approaches to mitigate the salt impacts on Soybean. Therefore, it is necessary to use more advanced biotechnological and breeding approaches to develop salt resilient Soybean germplasm.

Under salt stress, plants accumulate various organic osmolytes, including proline, glycine betaine, polyamines and sugar alcohols. These osmolytes maintain the intracellular osmotic potential to minimize the harmful effects of salinity stress (**Figure 5**). Therefore, osmolytes mediated interactive regulatory mechanism along with metabolites, antioxidants and phytohormones will be crucial to understanding soybean salinity tolerance mechanism. Many soybean salt stress-related studies have reported the activation of the proline synthesize gene, Δ1-pyrroline-5-carboxylate synthetase 1 (P5CS1) and subsequently, higher accumulation of proline. Proline has an osmotic adjustment capability and plays a protectant role in response to salt stress (Deinlein et al., 2014). Plant metabolism helps proline accumulation, which correlates with plant salt stress response and tolerance (Ahmad et al., 2016a; Nahar et al., 2016). However, recent studies suggested that proline can scavenge only hydroxyl radical but cannot scavenge superoxide, singlet oxygen, nitric oxide, nitrogen dioxide, and peroxynitrite, which puts proline role as a specific ROS scavenger under salt stress conditions (Nahar et al., 2016; Signorelli, 2016; Ahmad et al., 2016b). Furthermore, proline effects are dependent on concentrations, duration and developmental stage of plants under salt stress conditions (Mansour and Ali, 2017). In soybean, overexpression of *GmMYB46* induced the expression of salt stress response genes (*P5CS1*, *POD*, *SOD*, and *NCED3*), thus enhancing the proline and antioxidants accumulation for salt stress tolerance (Liu et al., 2021c). Sugar transport and distribution improve growth and development and stimulates plant salt stress response. Salt-tolerant varieties adjusted their cytoplasmic compatible solutes pool to deal with high salinity levels. Trehalose serves as cells’ metabolic resources and structural components and exhibits hormone-like regulating properties (Sarkar and Sadhukhan, 2022). Sugar synthesis genes, including trehalose-phosphate synthase (TPS), are involved in trehalose production and salt induced osmotic stress adjustment. Under salt stress, trehalose act as a signalling molecule, stabilizes dehydrated enzymes, proteins, and lipid membranes, and activates stress responsive genes for better osmotic stress adaptation in soybean (Cadavid et al., 2020b). Similarly, salt stress induces the sucrose synthesis genes and aromatic amino acids (precursor for phenolic compounds) production. Sucrose helps to balance the osmotic adjustment, and phenolic compounds are potent ROS scavengers in soybean (Noor et al., 2022). Exogenous glycine betaine (GB) application had the otoprotective role and act as a ROS scavenger. GB is an important osmolyte that improves Na^+^ homeostasis to enhance salt stress tolerance. GB has an excellent role in improving photosynthetic efficiency and antioxidant enzyme activity to mitigate salt-induced growth inhibition. Moreover, GB maintained the Na^+^/K^+^ ratio mainly by plummeting the buildup of Na^+^ in plants (Zhu et al., 2022). GB reduces the membrane peroxidation and improves the photosynthesis and yield under stress conditions in soybean (Khalid et al., 2022). GB treatment increased Na^+^ efflux in leaf protoplasts and reduced the cellular distribution of Na^+^. Furthermore, GB improved the vacuolar activity of NHX and V type H^+^-ATPases genes to improve salt tolerance (Zhu et al., 2022). A previous study reported that GB biosynthesis in plant tissues is highly energy intensive (Bai et al., 2022). Therefore, exogenous spraying of GB is more economic to enhancing soybean salt tolerance. Melatonin has many functions, including omo-protectant, seed priming agent, phytohormone regulator (auxin levels), ion homeostasis, and secondary messengers under abiotic stress. Exogenous application of melatonin reduces the salt effects by increasing the RWC, antioxidant activity, photosynthetic efficiency, cell division, carbohydrates, fatty acids and ascorbate content in soybean (Wei et al., 2015). Recently, a comparative study of GB and melatonin reported that GB is more efficient in relieving the salt effects in *Dalbergia odorifera* (Cisse et al., 2021). Such kind of studies are lacking in soybean. Therefore, a comparative study that includes a combined effects of both GB and melatonin and the right dose of exogenous spraying will be more beneficial to impart salt tolerance in soybean.

## Plant hormones and their cross-talk with ABA master regulator of salt stress tolerance

**Figure 6:**
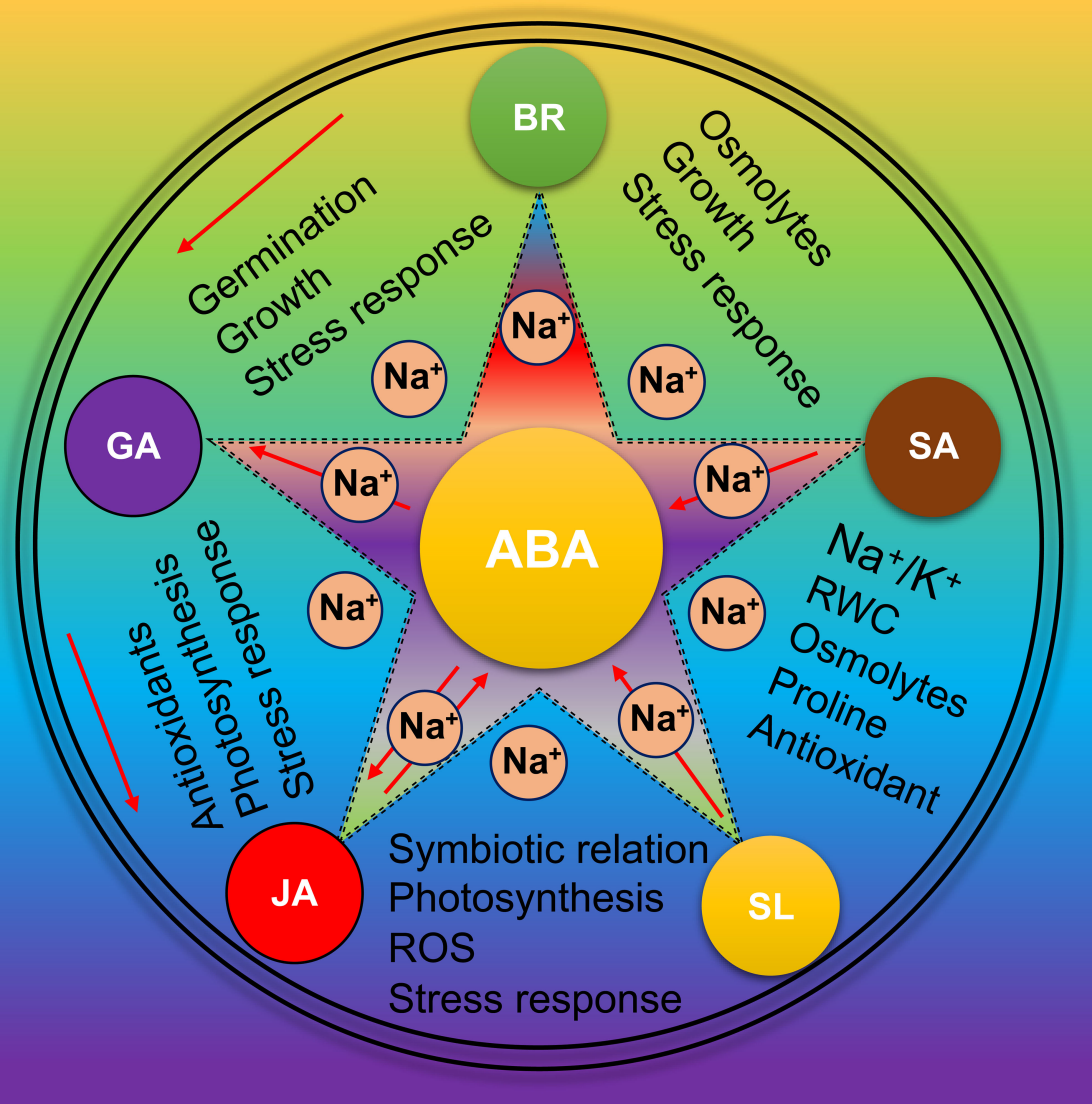
Role of phytohormones in Soybean salt tolerance Under salt stress, various phytohormones naturally activate in Soybean. These phytohormones work in a highly complex interactive network and has cross-talk with each other especially with ABA to reduce the impact of salt stress.

Different studies have explained that each plant hormone plays multiple biological roles depending on growth stages, tissues, and environmental conditions (Ku et al., 2018; Yu et al., 2020; Zaid et al., 2021). Here, we summarize how different hormones regulate or ameliorate the salt effects and help to optimize growth, adaptation and final production in soybean (**Figure 6**). BR is involved in plant adaptation to abiotic stresses. *BRI1-EMS suppressor 1* (*BES1*)/*brassinazole-resistant 1* (*BZR1*) regulates plant signalling pathways in soybean. In soybean, *BES1/BZR1 homolog 1* (*GmBEH1*) interacts with *GmBIN2*, a putative *BR-insensitive 2* (*BIN2*) to regulate the BR signalling (Yan et al., 2018). Soybean seed priming with BR (24-epibrassinolide, EBL) and nitrogen (N) nutrient solution improved salt stress tolerance. EBL and N triggered the accumulation of different osmolytes such as sugars, proline, and glycine betaine resulting in better protection by preserving tissue water content (Soliman et al., 2020). Therefore, the BR pathway has a cross-talk with osmolytes accumulators in soybean under salt stress. Further, some key enzymes involved in BR synthesis also affect plant salt adaption. For instance, *CYP85A1*-OE plants exhibited salt tolerance whereas *det2-1*, *SlDWARF*, *BRI1* or *BSK5* knockout plants were found salt-sensitive; however, exogenous BR application rescued the salt-sensitive phenotype of *det2-1* and *SlDWARF* (Cheon et al., 2010; Li et al., 2012; Zhu et al., 2016; Duan et al., 2017). Br also has a cross-talk with ABA; ABA inhibits BR signalling to regulate salt tolerance (Zhang et al., 2009). For example, *BRI1* or *BSK5* mutants were found sensitive to ABA and BR antagonized the ABA effects for modulating the salt tolerance (Zeng et al., 2010; Li et al., 2012). However, how these pathways respond to soybean salt tolerance is still elusive. Similarly, molecular evidence suggested that a close loop interaction mechanism between SA, GA and BR was activated in response to salt stress, thus conferring plant salt tolerance (Divi et al., 2010; Wang et al., 2019c). These studies suggested a direct or indirect involvement of BR signalling components in salt stress signals through modulation at transcriptional and post-translational levels. Finally, BRs have been proven an essential regulator of plant salt tolerance.

In response to salt stress, activation of JA signaling caused primary root growth inhibition (Zhao et al., 2014). However, *jaz3-1* and *jasmonates insensitive3* (*jai3*) mutant increased growth and root cell growth under salt stress (Valenzuela et al., 2016). These results depicted the involvement of the JA pathway in plant salt response. Exogenous JA treatment reduced the salt toxicity by ameliorating the ROS or ion homeostasis and interacting with different phytohormones (Farhangi-Abriz and Ghassemi-Golezani, 2018). Soybean seeds priming with JA, foliar application and/or their combination improved the various physiological traits such as water potential, water use efficiency, osmotic potential, RWC, and photosynthetic rate under salinity stress. JA treatments also improved ABA and GA phytohormones and stress-responsive genes expression of FeSOD, POD, CAT, and APX family under salt stress in soybean. Therefore, JA treatment could efficiently protect seedlings, alleviate salt stress damage, and improve soybean growth against excessive soil salinity (Sheteiwy et al., 2021). Interestingly, JA has a cross-talk with ABA. JA mutant plant demonstrated less accumulation of ABA, while the *JAZ1* gene was inducible by ABA treatment to promote salt tolerance (Brossa et al., 2011). Strigolactone (SL) roles in fungi-plant symbiotic interaction, growth and salt tolerance have been well established (Zhang et al., 2015a). For instance, exogenous GR24 enhanced plant growth by increasing the photosynthetic capacity and removing the salt stress effects (Ma et al., 2017). Similarly, exogenous 0.5 μM SL treatment could improve the alkaline and salt tolerance of soybean seedlings by managing the MDA and H_2_O_2_ levels and improving the antioxidant system and phenylpropanoid biosynthetic pathway. Furthermore, SL treatment promotes Na^+^ transport from the roots to the leaves of soybean seedlings to enhance salt tolerance (Chen et al., 2022). Salt treatment induced the SL biosynthesis-related carotenoid cleavage dioxygenases (CCD7 and CCD8) and MAX2 *via* an ABA-dependent way and upregulating the SL signaling pathway (An et al., 2016; Wang et al., 2019b). SL treatment also improved the salt tolerance of ABA mutants. These results also indicate a crosstalk between SL and ABA for salt tolerance execution in plants. However, exact mechanism of SL and ABA crosstalk yet need to be explored in soybean.

Salt stress reduces the bioactive GA levels and slows seed germination and plant growth (Zhu et al., 2015). Reduction of growth seems to be an adapted mechanism in response to lower GA to prepare the plant for salt tolerance. For example, Arabidopsis and rice GA metabolism-related genes such as GA2ox7, GA2ox5, OsDSK2a and MYB91 enhanced plant salt tolerance by hampering plant growth (Magome et al., 2008; Shan et al., 2014; Zhu et al., 2015; Wang et al., 2020a). Similarly, in soybean, salt stress delays seed germination by negatively regulating gibberellin (GA) while enhancing the ABA biogenesis, which causes an imbalance in the GA/ABA ratio. Fluridone (FLUN), is an inhibitor of ABA biogenesis that promote soybean seed germination under salt stress by altering the GA1/ABA, GA3/ABA, and GA4/ABA ratios (Shu et al., 2017). The cotton gene *GhPLATZ*-OE in Arabidopsis speeds up germination by suppressing the ABI4 (Zhang et al., 2018). The *ABI4* gene mediated the transcription of *GA2ox7* (GA catabolic) and the ABA synthesis gene *NCED6* (Shu et al., 2016). Higher GA is required for seed germination but lowered GA levels for salt stress tolerance. Therefore, it is direly necessary to identify a feedback loop mechanism to switch on or off the GA levels at the germination and later growth stages.

Salicylic acid (SA) is an essential phenolic compound that improves plant growth and defense systems under salt stress by accumulating osmolytes and an enhanced antioxidant system (Filgueiras et al., 2019; Dawood et al., 2022). SA also facilitates nitrogen fixation and microbial symbiotic relationships to enhance photosynthesis and productivity under salt-stress conditions (Ahanger et al., 2019). SA acts in a dosage manner and *snc1*, *sid2*, and *npr1-5* mutant plants were salt-sensitive (Xu et al., 2010; Jayakannan et al., 2015). Exogenous application of SA induced the proline accumulation through activation of *P5CS* and involved *MYB* genes in maintaining higher ABA levels to protect the salt stress damage (Zheng et al., 2018). Interestingly SA reduces lipid peroxidation and interacts with endogenous GAs and ABA to regulate the Na^+^/K^+^ balance to enhance salt tolerance (Liu et al., 2022b). It was reported that an exogenous supply of SA improves salt tolerance, growth and yield in soybean. SA enhanced the RWC, osmolytes, protein accumulation, enzymatic and non-enzymatic antioxidants (APX) and ionic homeostasis to enhance salt tolerance in soybean (Nigam et al., 2022). SA also regulated cellular Na^+^/K^+^ homeostasis through SA-mediated ionic channels to enhance salt tolerance and interactions with other plant hormones like Cytokinin and GA (Liu et al., 2019c).

## Natural variation for salt stress tolerance in soybean

During domestication, artificial selection caused the loss of important stress-responsive genetic loci in cultivated soybean. A significant variation exists among cultivated and wild soybeans for the salt response. The allelic diversity and beneficial alleles could be incorporated into domesticated soybeans to improve salt tolerance. Therefore, identifying natural alleles controlling salt tolerance has great potential to improve the soybean yield through genetic improvement. Recently, *Glyma03g32900* (*GmCHX1/GmSALT3*) was identified as a novel shoot sodium ion transporter and associated with salt tolerance (Guan et al., 2014; Qi et al., 2014). *GsCLC‐c2* squeezed excessive Cl^−^ into the vacuoles of root cells to improve salt tolerance (Wei et al., 2016). A recent genome-wide study identified 34 salt‐tolerant wild soybeans. A 7‐bp insertion/deletion (InDel) in the promoter of *GsERD15B* (early responsive to dehydration 15B) regulates the salt tolerance differences in soybean germplasm. *GsERD15B* contained a PAM2 domain for interaction with poly(A)‐binding (PAB) proteins. The 7‐bp deletion caused up‐regulation of *GsERD15B*, two *GmPAB* genes, and various stress‐related genes, including *GmABI1*, *GmABI2*, *GmCAT4*, *GmbZIP1*, *GmP5CS*, *GmPIP1:6*, *GmSOS1* and *GmMYB84* in response to salt stress. Natural variation in *GsERD15B* promoter and overexpression of *GsERD15B* increased the ABA‐signalling, proline content, catalase peroxidase, dehydration response and cation transport to enhance salt tolerance in soybean (Jin et al., 2021b). Natural variations were also identified for dehydration-responsive element-binding (DREB) family transcription factor genes in cultivated soybean. Recently, GWAS identified 103 DREB genes by combining with RNA-sequencing and population genetics of wild, landrace, and cultivated soybean accessions. DREB3a underwent artificial selection, but the DREB3b allele (DREB3b39Del) contributed to salt tolerance in soybean (Hou et al., 2022). Natural variation in gene promoter region also contributes towards salt tolerance in soybean. For instance, four promoter haplotypes of a class B heat shock factor HSFB2b were identified, which repressed the *GmNAC2* to improve flavonoid accumulation by activating the flavonoid biosynthesis pathway genes to enhance salt tolerance. Promoter haplotype II was selected during early domestication than haplotype III and had a low distribution frequency (Bian et al., 2020b). Similarly, SNP-based GWAS helped to locate the *Glyma03g32900* gene on Chr 3 based on three significant SNP markers, Salt-20, Salt14056 and Salt11655, which were highly correlated with salt-related traits. Additionally, a new minor locus on Chr 8 was also predicted for salt tolerance in soybean (Do et al., 2019b). These results suggested that natural variations exist in highly diverse soybean germplasm, which could be exploited for salt tolerance.

## Enhancement of salt tolerance in soybean using QTL-omics approach

Marker-assisted breeding (MAB) is an efficient approach for developing salt-tolerant soybeans. MAB helps to capture the natural variations in chromosomal regions, termed as QTLs. Major QTLs that contributed to salt tolerance are important for breeding. MAB uses DNA markers associated with particular QTLs that help identify genotypes with the desired alleles (Abbas et al., 2022; Zhang et al., 2015b). Recently, MAB helped to identify a major QTL qST-8 related to salt tolerance which is closely linked with the marker Sat_162 and detected on Chr 8 using a population of 184 recombinant inbred lines (RILs). Similarly, GWAS identified several SNPs in the same genetic region on Chr 8, which were significantly associated with salt tolerance. A candidate gene *Glyma.08g102000* (*GmCDF1*) belong to the cation diffusion facilitator (CDF) family was identified in this region. *GmCDF1* affects the K^+^/Na^+^ ratio and negatively regulates salt tolerance by regulating the expression of two ion homeostasis-associated genes, *GmNHX1* and *GmSOS1*, in transgenic hairy roots (Zhang et al., 2019a). Similarly, another study identifies major salt-related loci on Chr 3 and Chr 13 using a mapping population of 132 F2 families derived from Williams 82 (salt sensitive) and Fiskeby III (salt tolerant). On Chr 3, three genes were identified based on polymorphic markers, Salt-20, Salt14056, and Salt11655, and significantly associated with salt tolerant phenotype (Do et al., 2018). Similarly, using a RIL population derived from salt-tolerant (Jidou 12) and the salt-sensitive (Ji NF 58) identified a major salt-tolerant QTL, flanked by SSR markers GMABAB and Barcsoyssr_03_1421 on Chr 3, might be useful in marker-assisted selection for soybean salt tolerance (Shi et al., 2018b). A mapping population descendent from a cross between a salt-sensitive Cheongja 3, and a salt-tolerant landrace, IT162669, was analyzed for QTLs conferring salt tolerance. Two novel major loci, qST6, on Chr 6, and qST10, on Chr 10, and eight candidate genes involved in phosphoenolpyruvate carboxylase and an ethylene response factor that control ion toxicity and physiology in response to salt were identified (Cho et al., 2021). A F2:3 mapping populations, was used for QTL mapping, derived from NY36-87 and two salt-sensitive Zhonghuang39 and Peking soybean cultivars. A salt tolerance locus on Chr 3 and Chr 18 that harbored *GmSALT3* and *GmSALT18* genes co-segregated with the salt tolerance locus was identified using simple sequence repeat SSR markers and bulked segregant analysis (BSA) mapping strategy. This study provides genetic material and novel loci for salt-tolerant soybean breeding (Guo et al., 2021).

## Progresses of transcriptomic of soybean under salt stress

Transcriptome analysis is a powerful approach to exploring genome-wide gene expression reprogramming in response to salt stress. The expression and function of genes controlling physiology and metabolism must be modified for plants to endure and recover from salt stress. Recently, under salt stress, RNA-seq analysis identified 15,997 and 15,494 DEGs in the leaves and roots of soybean. The DEGs enrichment analysis divided molecular pathways into different adaptation-related processes under salt stress. The leaf enriched DEGs modules were related to amino acid, protein turnover, carbon fixation, photosynthetic process, phytohormone, and primary nitrogen metabolism. In contrast, root-enriched DEG modules were starch and sucrose metabolism, phenylpropanoid biosynthesis, cell wall and cell membrane composition reactive oxygen species (ROS) scavenging (Liu et al., 2019a). Similarly, another transcriptome analysis revealed 1,235 DEGs under salt stress. Out of a total of 116 TFs, 17 belonged to MYB families. Functional analysis revealed that *GmMYB46* localized in the nucleus, up-regulated by salt and mannitol, and enhanced the salt tolerance by ectopic-overexpressing of *GmMYB46* in Arabidopsis. Although, *GmMYB46* upgraded the expression of salt stress response genes (*P5CS1*, *SOD*, *POD*, and *NCED3*), but actual mechanism is still vague in soybean and Arabidopsis (Liu et al., 2021c). Transcriptomic analysis of the salt-tolerant ‘Qi Huang No.34’ (QH34) and the salt-sensitive cultivar ‘Dong Nong No.50’ (DN50) identified 17,477 genes responsive to salt stress. Among these, 6644 were DEGs between the two soybean cultivars. These DEGs were enriched in different stress-responsive pathways like phenylpropanoid biosynthesis, phytohormone signalling, the mitogen-activated protein kinase pathway, ribosome metabolism and oxidoreduction, which may play crucial roles in response to salt stress (Hu et al., 2022). The transcript expression profile of a drought-sensitive (C08) and a tolerant (Conquista) after 4 h of salt stress identified 1400 DEGs. Among them, 647 were induced, and 753 were repressed. These DEGs were enriched in ABA, BR signalling pathways, ethylene biosynthesis, DNA repair, and the plastid translation process (Cadavid et al., 2020b). Transcriptomic analysis of RA-452 x Osage mapping population, along with two F4:6 lines with salt tolerant and sensitive lines a total of 2374, 998, 1746, and 630 DEGs in a time series experiment, at 0, 6, 12, and 24 h, respectively. This study identified 13 common DEGs; six were up and seven DEGs were downregulated in the salt-tolerant line. This study reported key potential genes involved in the salt-tolerance such as *Glyma.02G228100*, *Glyma.03G226000*, *Glyma.03G031000*, *Glyma.03G031400*, *Glyma.04G180300*, *Glyma.04G180400*, *Glyma.05g204600*, *Glyma.08G189600*, *Glyma.13G042200*, and *Glyma.17G173200* in the soybean salt-tolerant line (Zeng et al., 2019).

## Role of metabolome in soybean salt stress tolerance

Metabolites are low-molecular-weight compounds produced in plants. These metabolites proved to be defensive compounds against biotic and abiotic stresses in plants. During their evolution, plants synthesize new metabolites, and about 200,000 specialized metabolites are produced by plants (Sugiyama, 2019). Salt stress leads to osmotic stress and changes the metabolomic state in soybean. Recently a proteomics study identified salt stress-responsive phosphoproteins in soybean. Upon salt treatment, out of a total of 4698 phosphopeptides, 412 were significantly up-regulated. *GmMYB173* protein was differentially phosphorylated at serine 59 upon salt treatment and acted as a substrate for the casein kinase-II. Phosphorylation of MYB binding sites in the promoter of *GmCHS5* (flavonoid synthase gene) facilitated *GmMYB173* binding. Metabolomics analysis of *GmCHS5* catalyzed chalcone and flavonoids revealed that 24 flavonoids of 6745 metabolites were significantly up-regulated upon salt treatment. Overexpression of *GmMYB173S59D* and *GmCHS5* resulted in the accumulation of cyaniding-3-arabinoside chloride belonging to dihydroxy B-ring flavonoid (an antioxidative agent) that enhanced the soybean salt tolerance. Therefore, salt stress-mediated phosphorylation of *GmMYB173* increased its binding affinity to the *GmCHS5* promoter. Thus, the *GmMYB173-GmCHS5* module contributed to the accumulation of dihydroxy B-ring flavonoids and enhanced soybean salt tolerance (Pi et al., 2018). Metabolic profiling of soybean plants revealed that the TCA cycle and amino acid metabolism contributed to the salt response. Soybean varieties with higher nitrogen absorption, assimilation rate, faster TCA cycle activity and more amino acid accumulation for better salt stress response. Rhizospheric secreted soil microbes rapidly degrade metabolites to mediate biological communication. Isoflavones and strigolactones metabolites help to develop a symbiotic relationship between rhizobia and arbuscular mycorrhizal fungi and soybean root (Massalha et al., 2017; Mishra et al., 2022). These symbiotic relationships also help to improve the salt tolerance in soybean.

## Functional genomics of salt responsiveness in soybean and transgenic technology

**Figure 7:**
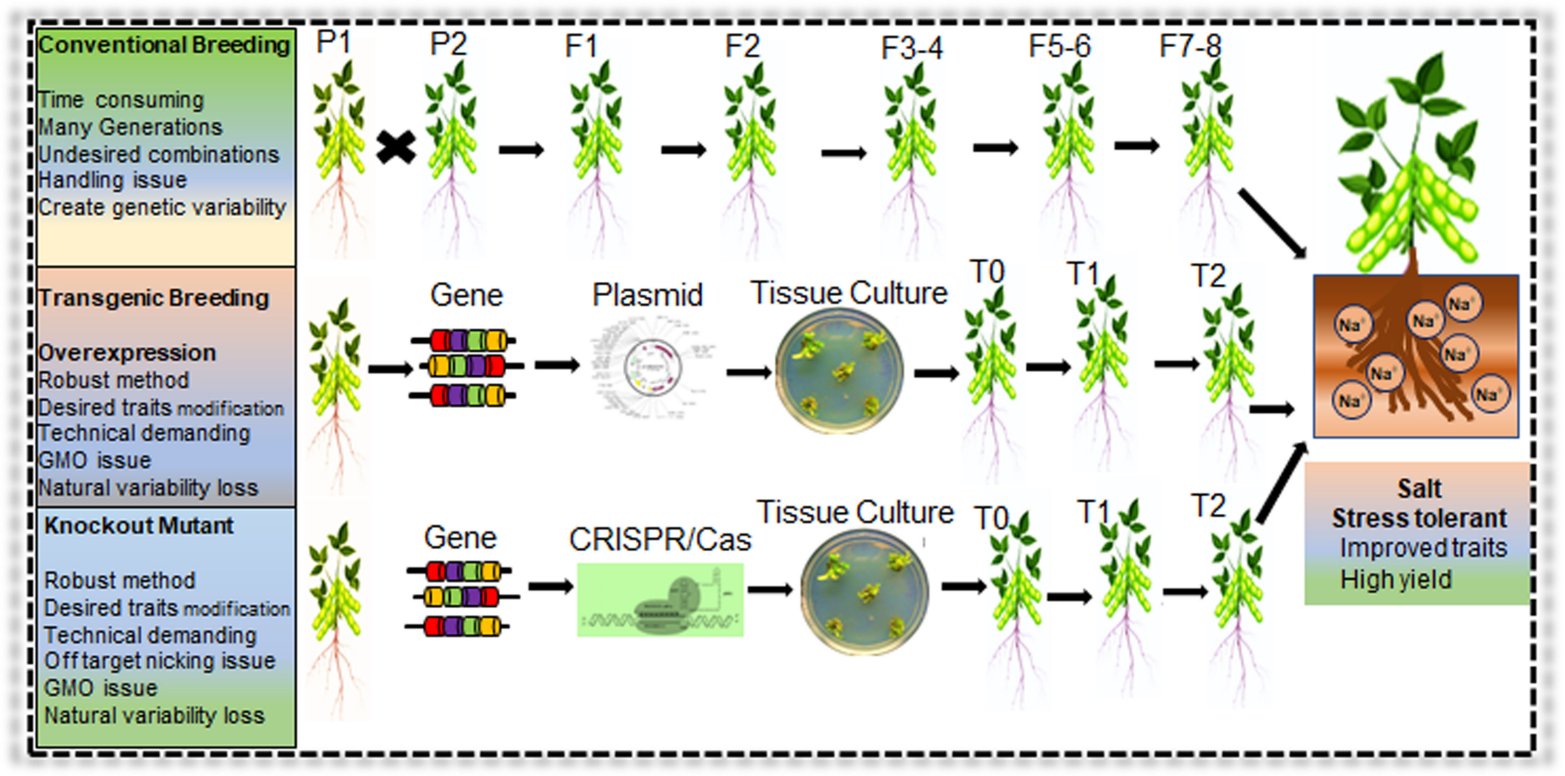
Conventional and modern genetic approaches for development of salt stress tolerant Soybean. Application of conventional breeding, transgenic breeding, and genome editing (CRISPR/Cas) system for salt tolerant Soybean germplasm development. Modern biotechnology approaches are robust that can generate stress responsive germplasm in a short duration compared to conventional breeding methods.

In planta, genes functional validation is necessary to identify the role of a particular gene in salt tolerance in soybean. Due to the highly complex genome structure of soybean, in the last two decades, soybean genetic transformation progress remained slow and inefficient. Therefore, functional gene validation was primarily performed in Arabidopsis or transient expression in soybean hairy root (**Table 2**). Several soybean genetic transformation protocols were developed with variable success rates, such as immature cotyledons, hypocotyls, shoot meristems, embryos, half-seed explants and cotyledonary nodes (Zhang et al., 2022b). Soybean Agrobacterium-mediated cotyledonary node (CN) transformation has an average transformation efficiency of 3.8%–8.7% (Li et al., 2017). Recently, some studies reported soybean average transformation efficiency up to 18.7% but still lower than rice and maize (Pareddy et al., 2020; Zhang et al., 2022b). Many factors combinedly affect the soybean transformation efficiency, including varieties, Agrobacterium medium concentration, hormone levels, explant co-cultivation time and explant regeneration capability (Li et al., 2017). Compared with conventional breeding, transgenic approaches have many advantages, including short duration, targeted genome modification and traits improvements (**Figure 7**). For instance, if transgenic approaches are coupled with speed breeding, the advanced elite germplasm creation process will be more robust and readily available for large-scale cultivation (Hussain et al., 2023).

**Table 2:** Summary of functional validation of salt related genes in Soybean.

Gene	Transgenic	Functions	Regulation	Remarks	Reference
GmLecRlk	Soybean	ROS Antioxidant GmDREB2 GmERF3 GmbHLH30 GmLAMP1 GmGH3.6 GmPUB8	Down UP UP UP UP UP Down Down	Salt tolerance	Zhang et al., 2022c
GmVOZ1G	Soybean	Antioxidants RWC	UP UP	Salt tolerance	Li et al., 2020
GsPRX9	Soybean	Root POD SOD Glutathione Enzymes activity	UP UP UP UP UP	Salt tolerance	Jin et al., 2019
GmCIPK21- GmCBL4	Soybeans	ROS ABA	Down Up	Salt tolerance	Li et al., 2022a
GmPKS4	Soybeans	Osmolyte ROS Stress gene	UP Down UP	Salt tolerance	Ketehouli et al., 2021
GmSIN1- GmNCED3s- GmRbohBs	Soybean	ABA ROS Root Yield	UP Down UP UP	Salt tolerance	Li et al., 2019c
GmNAC085	Soybean	Germination Antioxidant Proline Dehydrins genes	UP UP UP UP	Salt tolerance	Hoang et al., 2021
GmMYB118	Soybeans	Osmotic Oxidizing Stress genes	Down Down UP	Salt tolerance	Du et al., 2018
GmbZIP2	Soybean	GmDHN15 GmGST1 GmMYB48 GmWD40 GmLEA	UP UP UP UP UP	Salt tolerance	Yang et al., 2020
GmbZIP15	Soybean	GmWRKY12 GmABF1 GmSAHH1	Down Down UP	Salt sensitive	Zhang et al., 2020a
GmTLP8	Soybean	Stress genes	UP	Salt tolerance	Xu et al., 2022b
GmDUF4228	Soybean	Leaf curling Wilting MDA H_2_O_2_ O^2-^ RWC Proline Chlorophyll	Down Down Down Down Down UP UP UP	Salt tolerance	Leng et al., 2021
GmPI-PLC7	Soybean	Chlorophyll O^2-^ H_2_O_2_ NOX	UP Down Down Down	Salt tolerance	Chen et al., 2021
GmC2-148	Soybean	Proline H_2_O_2_ O^2-^ Down MDA Down Stress-genes	UP Down Down Down UP	Salt tolerance	Sun et al., 2021b
GmCDPK3	Soybean	Membrane damage MDA Proline Chlorophyll	Down Down UP UP	Salt tolerance	Wang et al., 2019a
GmCBP60A-1	Soybean	Proline Electrolyte leakage MDA	UP Down Down	Salt tolerance	Yu et al., 2021
GmMYB81- GmSGF14l module	Soybean	Germination Chlorophyl	UP UP	Salt tolerance	Bian et al., 2020a
GmTIFY10e/g	Soybean	Proline POD CAT MDA ABA GmSnRK2 GmCAT1 GmPP2C GmPOD	UP UP UP Down UP UP UP UP UP	Salt tolerance	Liu et al., 2022a
GmTGA17	Arabidopsis and Soybean	Chlorophyll MDA Proline	UP Down UP	Salt tolerance	Li et al., 2019a
GmSAP16	Soybean	Proline Chlorophyll MDA Down GmDREB1B;1 GmRD22 UP GmDREB2 GmNCED3 GmNHX1 GmSOS1 UP	UP UP Down UP UP UP UP UP UP	Salt tolerance	Zhang et al., 2019b
GmFAD3A	Soybean	JA UP Chlorophyll RWC Proline GmWRKY54	UP UP UP UP UP	Salt tolerance	Singh et al., 2022
GmNAC109	Arabidopsis	DREB1A DREB2A AREB1 AREB2 RD29A COR15A AIR3 ARF2	UP UP UP UP UP UP Down Down	Salt tolerance	Yang et al., 2019
GmEF4	Soybean	Proline H_2_O_2_ O^2-^ MDA	UP Down Down Down	Salt tolerance	Gao et al., 2019
GmGRAS37	Soybean	Salt genes	UP	Salt tolerance	Wang et al., 2020b
GmCrRLK1L20	Soybean	GmDREB-like GmMYB84 GmGST15 GmWRKY40 GmNAC29 GmbZIP78	UP UP UP UP UP UP	Salt tolerance	Wang et al., 2021b
GmPP2A-B-71	Soybean	Stress genes ROS	UP Down	Salt tolerance	Xiong et al., 2021
GmDREB6	Soybean	GmP5CS Proline	UP UP	Salt tolerance	Nguyen et al., 2019
GmNAC06	Soybean	Na^+^/K^+^ ratio GmUBC2 GmHKT1	UP UP UP	Salt tolerance	Li et al., 2021
GsCLC-c2	Soybean & Arabidopsis	Ionic homeostasis	UP	Salt tolerance	Liu et al., 2021b
GmTGA13	Arabidopsis & Soybean	Stress genes K^+^ Ca^2+^	UP UP UP	Salt tolerance	Ke et al., 2022
GmMYB68	Soybean	Osmotic adjustment Photosynthetic Yield	UP UP UP	Salt tolerance	He et al., 2020b
GmELF3	Soybean	GmNAC GmSIN1 UP GmWRKY12 GmWRKY27 GmWRKY54	UP UP UP UP UP	Salt tolerance	Cheng et al., 2020
GmWRKY12	Soybean	Proline MDA	UP Down	Salt tolerance	Shi et al., 2018a
GmANK114	Soybean	Proline ROS WRKY13 NAC11 MYB84 DREB2 bZIP44	UP Down UP UP UP UP UP	Salt tolerance	Zhao et al., 2020
AtABF3	Soybean	Ion leakage Chlorophyll	Down UP	Salt tolerance	(Kim et al., 2018
AtARA6	Soybean	MYC2 WRKY6 WRKY86 Inositol oxygenase	Down Down Down UP	Salt tolerance	Hong et al., 2022
GmbZIP19	Arabidopsis	Stomatal damage Stress gene	UP Down	Salt sensitive	He et al., 2020a
GmG6PD7	Arabidopsis	Germination Root length ABA ROS	UP UP Down Down	Salt tolerance	Jin et al., 2021a
GsMYB15	Arabidopsis	Salt genes	UP	Salt tolerance	Shen et al., 2018
GmNHX1	Arabidopsis	Na^+^ K^+^/Na^+^ ratio	Down UP	Salt tolerance	Sun et al., 2019b
GmAKT1	Arabidopsis	K^+^/Na^+^ ratio Antioxidants	UP UP	Salt tolerance	Feng et al., 2021a

## Overexpression of soybean salt-responsive genes

Recently, transcriptome analysis identified *GmLecRlk* (*Glyma.07G005700*) a candidate salt-responsive gene in soybean. Overexpression of *GmLecRlk* in hairy root positively affected salt tolerance mainly through antioxidant activity and enhanced ROS scavenging ability in soybean. Furthermore, *GmLecRlk* improves the salt tolerance by upregulating *GmDREB2*, *GmERF3*, and *GmbHLH30* and downregulating *GmLAMP1*, *GmGH3.6* and *GmPUB8* of soybean (Zhang et al., 2022c). Zinc-finger (VOZ) transcription factors showed differential expression under dehydration, SA and salt stress conditions. Overexpression of *GmVOZ1G* in soybean hairy roots positively affected drought and salt tolerance by improving antioxidants and maintaining higher RWC. While RNA interference (RNAi) knockout *GmVOZ1G* soybean plants were sensitive to salt and drought stresses (Li et al., 2020). Modulation of antioxidant genes has positive effects on salt tolerance in soybean. Overexpression of the wild soybean peroxidase *GsPRX9* gene improved the morphological traits such as fresh root weight, primary root length, POD, SOD and glutathione enzymes activities, subsequently improving salt tolerance (Jin et al., 2019). Calcineurin B-like protein-interacting protein kinases (CIPKs) are involved in plant adaptation to abiotic stresses. Overexpression or knockout of *GmCIPK21* in soybean hairy roots led to increased or decreased salt tolerance, respectively. Further, *GmCIPK21* physically interacted with *GmCBL4* to scavenge salt-induced ROS and improved ABA response to improve salt tolerance (Li et al., 2022a). Similarly, another CIPK gene (*GmPKS4*) is upregulated under alkali, salt-alkali, drought, or ABA and localized in the nucleus and cytoplasm in soybean. Overexpressing GmPKS4 in Arabidopsis and soybean hairy roots enhances osmolyte accumulation, ROS scavenging, and salt stress-related gene regulation (Ketehouli et al., 2021). Overexpression of *SALT INDUCED NAC1* (*GmSIN1*) belonging to the *NAM/ATAF1/2/CUC2* (*NAC*) transcription factors promoted root growth, increased yield and salt tolerance in soybean. RNAi soybean for *GmSIN1* had the opposite effect. Furthermore, a positive feed-forward system enables the rapid salt signaling through *GmSIN1*, 9-cis-epoxycarotenoid dioxygenase coding genes (*GmNCED3*s) involved in ABA synthesis and *Respiratory burst oxidase homolog B* (*GmRbohBs*) associated with ROS generation which enables the rapid homeostasis of ABA and ROS signalling for salt tolerance (Li et al., 2019c). Under salt stress, transgenic soybean plants overexpressing *GmNAC085* displayed better germination rates due to higher activities of antioxidant enzymes and activation of key stress-responsive proline and dehydrins genes (Hoang et al., 2021). Overexpression of *GmMYB118* in soybean hairy roots improved drought and salt tolerance. While CRISPR-mediated knockout plants showed reduced drought and salt tolerance. *GmMYB118* improved drought and salt tolerance by regulating osmotic and oxidizing substances and promoting the expression of stress-associated genes (Du et al., 2018). Similarly, overexpression of *GmbZIP2* improved the plant resistance to drought and salt stresses through enhanced expression of the stress-responsive genes, including *GmDHN15*, *GmGST1*, *GmMYB48*, *GmWD40*, and *GmLEA* in soybean (Yang et al., 2020). Further, some TFs are negative regulators of soybean salt tolerance. For instance, overexpression of *GmbZIP15* caused hypersensitivity to salt stress in soybean mainly through repressing effects on stress-responsive genes involved in both ABA-dependent and ABA-independent pathways. *GmbZIP15* negatively regulates *GmWRKY12* and *GmABF1* and positively regulates *GmSAHH1* expression in response to abiotic stress (Zhang et al., 2020a). Overexpression of *GmTLP8* (Tubby-like proteins 8) improved salt and drought stresses tolerance, whereas *GmTLP8*-RNAi knockout promoted stress sensitivity in soybean (Xu et al., 2022b). Under salt stress, overexpression of *Domain of unknown function 4228-70* (*DUF4228*) in soybean reduced the leaf curling, wilting MDA, H_2_O_2_, and O^2-^ and increased the RWC, proline, and chlorophyll contents to enhance the salt tolerance (Leng et al., 2021). Phospholipase C (PLC) hydrolysis the phospholipids and is involved in plant stresses. soybean overexpressing the *GmPI-PLC7* exhibited drought and salt tolerance, while the *GmPI-PLC7*-RNAi lines exhibited sensitive phenotypes (Chen et al., 2021). Soybean C2 domain-containing proteins *GmC2-58*, *GmC2-88*, and *GmC2-148* responded to salt, drought, and ABA treatments. Transgenic soybean plants of *GmC2-148* maintained higher proline content, lower H_2_O_2_, O^2-^ and MDA and increased expression of stress-responsive genes, which enhanced drought and salt tolerance in soybean (Sun et al., 2021b). Calcium-dependent protein kinases (CDPKs) responded to a variety of abiotic stresses. Overexpression of *GmCDPK3* improved drought and salt tolerance compared with *GmCDPK3*-RNAi by reducing the cell membrane damage, MDA and accumulation of higher proline (Pro) and chlorophyll contents in soybean (Wang et al., 2019a). *Calmodulin-binding protein 60* (*CBP60*) is located in the cytomembrane and induced by drought and salt stresses. Soybean hairy roots overexpressing the *GmCBP60A-1* increased the proline content, lower electrolyte leakage and MDA, which likely enhanced the drought and salt tolerance, compared to *GmCBP60A-1*-RNAi soybean (Yu et al., 2021). A novel R2R3-type MYB (*GmMYB81*) differentially accumulated during embryo development, drought, salt and cold in soybean. *GmMYB81* interacts with the abiotic stress regulator *GmSGF14l* and cooperatively affects soybean abiotic stress tolerance and could be a candidate gene for salt stress tolerance development at seed germination (Bian et al., 2020a). Overexpression of *GmTIFY10e* and *GmTIFY10g* genes improved salt tolerance by increasing the PRO, POD, and CAT activity and decreasing the MDA content, contrasting to the *GmTIFY10e-GmTIFY10g*-RNAi plants which exhibited sensitivity to salt stress in soybean. Further analysis showed that *GmTIFY10e* and *GmTIFY10g* regulate the transcript levels of genes related to the ABA signal pathway, such as *GmSnRK2*, *GmMYC2*, *GmCAT1*, *GmPP2C* and *GmPOD* (Liu et al., 2022a). *GmTGA17*, expression was induced by both salt and drought stresses, is a nuclear-localized protein. Overexpression of GmTGA17 in Arabidopsis and soybean enhanced drought and salt stress tolerance. However, soybean *GmTGA17*-RNAi plants exhibited sensitivity to drought and salt stress. *GmTGA17*-OE improved the physiological parameters such as chlorophyll and proline contents and decreased the MDA content in soybean compared to RNAi plants (Li et al., 2019a). Similarly, A20/AN1 zinc finger domain containing stress-associated proteins (SAPs) *GmSAP16* gene was identified as a novel regulator of water deficit stress, salt, and abscisic acid (ABA) stresses. The overexpression of *GmSAP16* enhanced the drought and salt tolerance by modifying the proline and chlorophyll and lowering the MDA contents compared to *GmSAP16*-RNAi soybean seedlings. *GmSAP16*-OE and *GmSAP16*-RNAi also modified the expression of stress-related genes, including *GmDREB1B;1*, *GmRD22*, *GmDREB2*, *GmNCED3*, *GmNHX1*, and *GmSOS1* (Zhang et al., 2019b). Fatty acid desaturases (FADs) cause the desaturation of fatty acids by introducing double bonds and modulates membrane fluidity in response to abiotic stresses. Similarly, the overexpression of the Omega-3 Fatty Acid Desaturase in soybean showed higher accumulation of JA levels, chlorophyll content, RWC, proline content and higher expression of *GmWRKY54* as compared to *GmFAD3*-silenced plants and exhibited drought and salt tolerance (Singh et al., 2022). Similarly, overexpression of *GmNAC109*, a homologous of *AtAF1* in Arabidopsis, enhanced the drought and salt tolerance. *GmNAC109*-OE activated the expression of stress marker genes such as *DREB1A*, *DREB2A*, *AREB1*, *AREB2*, *RD29A*, and C*OR15A* in transgenic Arabidopsis. ABA-responsive genes *ABI1* (ABA INSENSITIVE 1) and *ABI5* were also induced, which caused hypersensitivity to ABA in transgenic Arabidopsis. However, *GmNAC109* could not induce the ABA-biosynthetic gene *NCED3* (N*INE-CIS-EPOXYCAROTENOID DIOXYGENASE 3*) expression and endogenous ABA content. However, *GmNAC109*-OE significantly increased the expression of *AIR3* (*AUXIN-INDUCED IN ROOT CULTURES 3*) and repressed the *ARF2* expression and lateral root formation in transgenic Arabidopsis lines (Yang et al., 2019). Soybean elongation factor 1α (EF1α) includes three structural domains: one GTP-binding domain and two oligonucleotide-binding domains that respond to drought and salt stress. *GmEF4*-OE soybean showed drought and salt tolerance through improved physiological traits and higher accumulation of proline and lower H_2_O_2_, O^2-^, and MDA (Gao et al., 2019). In soybean *GmGRAS37* was significantly induced by drought, salt, ABA and BR treatment. *GmGRAS37*-OE soybean plants exhibited improved resistance to drought and salt stresses *via* enhanced expression of drought and salt responsive genes (Wang et al., 2020b). The *Catharanthus roseus RLK1-like* (*CrRLK1L*) protein kinase *GmCrRLK1L20* responds to drought and salt stress. *GmCrRLK1L20*-OE improved drought and salt tolerance by activating stress responsive genes GmDRE*B-like*, *GmMYB84*, *GmGST15*, *GmWRKY40*, *GmNAC29* and *GmbZIP78* (Wang et al., 2021b). *Protein phosphatase 2A* (*PP2A*) regulates plants’ intracellular and extracellular ROS signals. Functional analysis demonstrated that *GmPP2A-B-71*-OE could improve drought and salt tolerance by activating stress responsive genes and ROS elimination in soybeans (Xiong et al., 2021). The DREBs gene belongs to the AP2 family. Overexpression of *GmDREB6* into *DT84* soybean cultivar enhanced the expression level of the *GmP5CS* gene and proline accumulation. Subsequently, transgenic soybean plants exhibited higher survival under salt stress treatment (Nguyen et al., 2019). Overexpression of *GmNAC06* (*Glyma.06g21020.1*) caused the accumulation of proline and glycine betaine and alleviating the ROS under salt stress. *GmNAC06*-OE regulates the Na^+^/K^+^ ratios by activating the *GmUBC2* and *GmHKT1* transporter to maintain ionic homeostasis and improve soybean salt tolerance (Li et al., 2021a). The *GsCLC-c2* gene and its promoter are activated in response to salt stress. In Arabidopsis or wild soybean plants overexpressing *GsCLC-c2*, the salt-induced growth reduction was markedly ameliorated through improved physiological traits compared to *GsCLC-c2*-RNAi wild soybean. *GsCLC-c2* regulated the anionic homeostasis in salt-stressed transgenic Arabidopsis and soybean, thus conferring enhanced salt tolerance (Liu et al., 2021b). *GmTGA13* induced the expression of the stress-responsive genes and absorption of K^+^ and Ca^2+,^ thus regulating the ion homeostasis in the cell (Ke et al., 2022). The MYB-TF involves plant development, secondary metabolism, and abiotic stress responses. *GmMYB68*-OE showed salt and alkali tolerance *via* osmotic adjustment and photosynthetic rates and improved yield compared to the *GmMYB68*-RNAi and WT (He et al., 2020b). The F-box family regulates salt responses and has a developmental role in soybean. At least 12 salt-responding F-box genes were identified in soybean (Jia et al., 2017b). *EARLY FLOWERING 3* (*ELF3*) gene suppressed salt stress response pathways to enhance salt tolerance. In soybean, the J allele is an ortholog of *AtELF3* Arabidopsis. Knockout mutants of J-alleles greatly prolong maturity and increase the soybean yield; however, reduced the salt tolerance. Overexpression of J-allele increased salt tolerance by positively regulating the expression of downstream salt stress response genes, including *GmNAC*, *GmSIN1* and *GmWRKY12*, *GmWRKY27*, *GmWRKY54* in soybean (Cheng et al., 2020). *GmWRKY12*-OE enhanced drought and salt tolerance *via* increased proline (Pro) content and decreased MDA of transgenic soybean (Shi et al., 2018a). *Ankyrin repeat* (*ANK*) proteins had multiple roles in plant growth and environmental stresses. Soybean G*mANK114* belongs to the RING finger (RF) domain-containing ANK-RF subfamily. *GmANK114*-OE improved the germination rate in Arabidopsis and soybean survival under drought and salt treatments. *GmANK114*-OE activated the transcription of abiotic stress-related genes *WRKY13*, *NAC11*, *DREB2*, *MYB84*, and *bZIP44*, under drought and salt stresses in soybean (Zhao et al., 2020). Stable soybean transgenic plants with ectopically expressing Arabidopsis *AtABF3* showed substantial salt stress tolerance by protecting the ion leakage rate and maintaining higher chlorophyll contents (Kim et al., 2018). Ectopic expression of *AtARA6* in Shen Nong 9 (SN9) soybean regulated the SNARE complexes in the vesicle transport pathway, which may enhance salt tolerance directly. In transgenic soybeans, *MYC2*, *WRKY6*, and *WRKY86* were downregulated while four inositol oxygenase genes were induced after salt treatment in transgenic soybeans (Hong et al., 2022). Ectopic over-expression of *GmbZIP19* in Arabidopsis increased the sensitivity to salt and drought by destroying the stomata and affecting the stress-related gene expression. *GmbZIP19* is a negative regulator of salt and drought stress tolerance (He et al., 2020a). Expression of Cytosolic *Glucose-6-phosphate dehydrogenase* (*G6PD* or *G6PDH*) and *GmG6PD7* were induced by NaCl treatment in soybean. Overexpression of *GmG6PD7* in Arabidopsis increased the seed germination rate, primary root length and markedly decreased the ROS levels in the transgenic plants. *GmG6PD7* affected the glutathione, NADPH and ABA levels and activates ROS scavengers to increase salinity tolerance in Arabidopsis. Complementation assay showed that *GmG6PD7* could rescue the seed and root phenotype of Arabidopsis cytosolic *G6PD* mutant (*Atg6pd5* and *Atg6pd6*) under salt stress (Jin et al., 2021a). *GsMYB15* from wild soybean is a typical R2R3-MYB TF that contains multiple stress-related cis-elements and is located in the nucleus. Arabidopsis plants, overexpressing *GsMYB15*, showed salt tolerance and enhanced resistance to *H. armigera* larvae. *GsMYB15* regulates transgenic plants’ defense and salt stress-related genes (Shen et al., 2018). Furthermore, the Ectopic expression of *GmNHX1* improved the morphology and generated more rosette leaves in Arabidopsis under salt stress conditions compared to the wild-type. *GmNHX1* increased the Na^+^ transportation to leaves and caused the reduction of Na^+^ absorption in roots. Therefore, *GmNHX1* maintained a higher K^+^/Na^+^ ratio under salt stress conditions (Sun et al., 2019b).

## Alternative splicing and soybean salt stress tolerance

**Figure 8:**
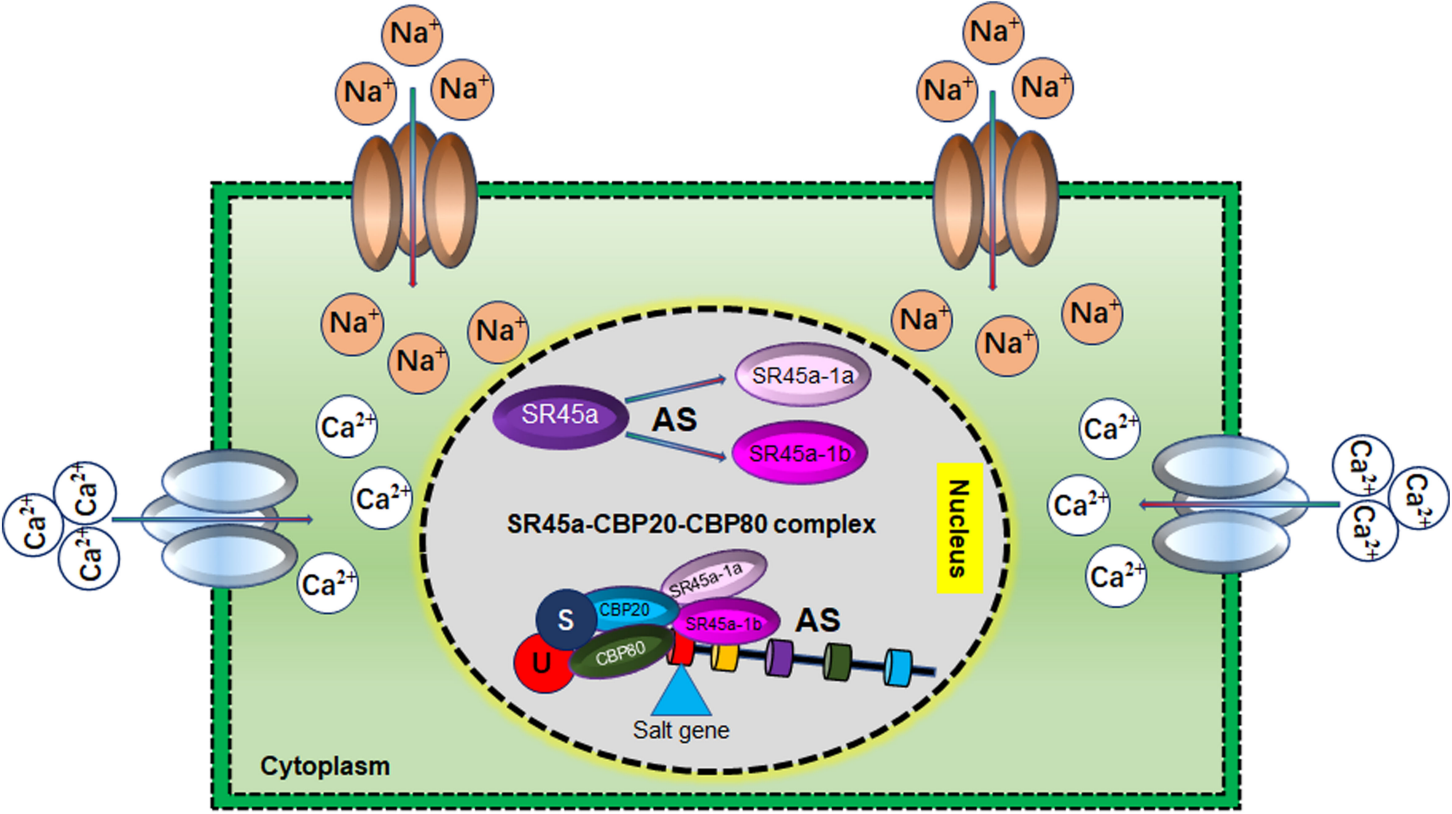
Role of alternative splicing (AS) in Salt tolerance Under salt stress conditions, AS of SR45a generates two variants SR45a-1a and SR45a-1b. These AS variants interact with CBP20 and CBP80 to form a regulatory cascade that regulates the alternative splicing of salt stress‐responsive genes. SR45a-1a and SR45a-1b regulate the salt tolerance in isoform-dependent manner.

Alternate splicing events could generate multiple mature mRNAs from one gene due to exon skipping, intron retention and AS of 5′ or 3′ sites (Ding et al., 2014; Laloum et al., 2018). The AS events could change genes’ protein structure, metabolic functions and cellular locations. Interestingly, AS variants could compete with normal gene transcript, thus enhancing protein function interference, transcriptome, proteome diversity and stress tolerance (Wani et al., 2020). A recent study has found that abiotic stress-responsive genes are more prone to AS (Yang et al., 2022). Salt stress promotes the alternative gene splicing, 5′ and 3′ splice-site selection introduces the premature termination codons (PTCs) in 49% of all intron-containing genes, thus generating multiple transcripts from the same allele (Ding et al., 2014). For instance, in soybean, salt stress produced the *ACYL-COA-BINDING PROTEIN* (*ACBP*) variants through AS, cannot interact with *lipoxygenases* (*LOXs*). Overexpression of *ACBP* AS-variants differentially regulates the expression of *LOXs* activity, lipid peroxidation and salt tolerance in soybean and Arabidopsis (Lung et al., 2022). Under salt stress, phytohormone ABA accumulates in the plant’s roots and is transported to leaves to regulate stomata or to avoid water loss. ABA also controls plant growth and improves salt stress tolerance through AS events of different transcription factors, protein kinases, and splicing factors. ABA-induced the AS mainly by increasing the number of unconventional splicing sites or by recruiting different splicing factors. ABA inhibits the *PP2C* phosphatases and activates *SnRK2* protein kinases to enhance the ABA signalling. The AS of the *HAB1* gene (a negative regulator of *SnRK2*) is regulated by ABA through intron retention and thus protects the inhibition of *SnRK2* protein kinases to respond to salt stress in an ABA-dependent manner (Wang et al., 2015b; Laloum et al., 2018). This evidence indicates that AS modulates salt stress responses by regulating the ABA-dependent and ABA-independent pathways, identifying the novel regulatory layer in plant salt stress tolerance. However, mRNA splicing is an essential cellular process; thus, it will be interesting to identify the regulatory components that transduce the signal to pre-mRNA splicing under salt stress and how they could be helpful for salt tolerance germplasm development. Recent studies have reported that in response to salt stress, AS of a serine/arginine‐rich splicing factor (SR) 45 produced two SR45.1 and SR45.2 variants. SR45.1 was found to be responsive to salt stress and rescued the salt-sensitive phenotype (**Figure 8**). Further, *SR45a*-*CBP20*-*CBP80* (c*ap‐binding protein 20*) cascade mediates the splicing of salt stress‐responsive genes by involving ubiquitination and sumoylation process (Kong et al., 2014; Albaqami et al., 2019; Li et al., 2021b). Thus, these studies show that the *SR45*‐*CBP20*-*CBP80* pathway is a critical regulator in pre‐mRNA splicing in response to salt stress (Wang et al., 2023).

## Epigenetic modification and epigenetic memories of salt stressed soybean

**Figure 9:**
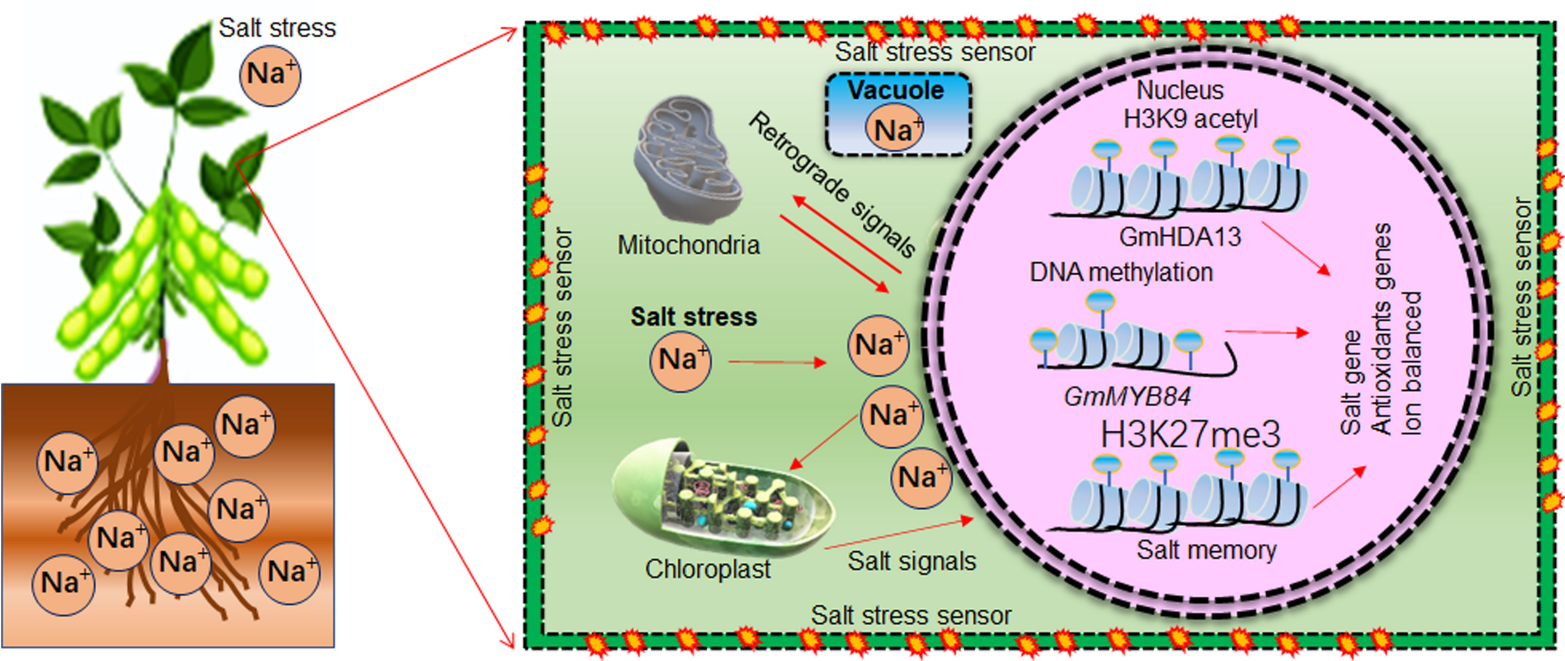
Role of epigenetics and retrograde signaling in Soybean salt tolerance Epigenetic modification alters the transcriptional landscape of salt stress responsive genes through methylation and acetylation. These changes are also a source of epigenetic memory that help to survive the plants under repertoire stresses. Retrograde signaling between different organs like chloroplast and nucleus helps salt homeostasis and activation of different stress-responsive pathways to reduce the salt effects in Soybean.

Although plants lack a nervous system but are intelligent; plants remember, choose, and make decisions to optimize their resources and enhance their fitness in response to abiotic stresses. Plants inherit this information to the next generation(s) through epigenetic modifications to adapt more efficiently to climate change (Gallusci et al., 2022). Plant memory is solely based on cellular, molecular, and biochemical networks such as metabolic, genetic, and epigenetic memories. Several epigenetic players, such as proteins or RNA, determine the plant epigenetic responses to stresses through histone modifications, DNA methylation, phosphorylation, ubiquitination and chromatin structure, thereby regulating gene expression (**Figure 9**). For instance, nuclear factor Y subunit *GmNFYA* mediated histone acetylation improved the salt tolerance by inducing the salt-responsive genes. *GmNFYA* interacts with *GmFVE*. *GmFVE* functions with histone deacetylase *GmHDA13* for transcriptional repression by reducing H3K9 acetylation at target loci. *GmNFYA* also competes with *GmHDA13* for interaction with *GmFVE*. This competition led to the activation and maintenance of histone acetylation for enhanced expression of salt-responsive genes and conferred salt tolerance in soybean (Lu et al., 2021). Similarly, DNA methylation played a critical role in responses to abiotic stress. Salt stress-mediated expression of *GmMYB84* relies on DNA methylation (**Figure 9**). *GmMYB84*-OE improved the plants traits such as germination rate, osmolytes and antioxidant enzyme activity and K^+^ levels under salinity stress. EMSA analysis revealed that *GmMYB84* physically binds to the cis-regulatory sequences of *GmAKT1*. Thus, DNA methylation modulates *GmMYB84* expression and thereby enhances the salinity stress tolerance of soybean (Zhang et al., 2020b). The ubiquitin/proteasome pathway also played a critical role in soybean development and salt tolerance. For example, over-expression of SENESCENCE-SUPPRESSED PROTEIN PHOSPHATASE (SSPP) severely suppressed normal plant growth but improved plant salt tolerance through ROS scavenging. The N-terminal 1-14 residues of ACS7 negatively regulate SSPP protein accumulation through the ubiquitin/proteasome pathway under normal growth conditions. However, ACS7-mediated SSPP protein degradation was repressed by salt and leaf senescence signals through the ubiquitin/proteasome pathway, which providing an effective strategy for salt-tolerant soybean breeding (You et al., 2022). Similarly, histone marks modifications are important for epigenetic memory under salinity stress in soybean. Primed soybean seedlings exhibited a different transcriptomic landscape than non-primed soybean seedlings, indicating some kind of epigenetic memory. Priming induced alterations in histone marks, histone 3 lysine 4 dimethylation (H3K4me2), H3K4me3, and histone 3 lysine 9 acetylation (H3K9ac) coordinately altered the salt stress response through modification ion homeostasis, cell wall and defense-related transcriptional network. Further chemical inhibitors could alter the histone acetylation status and elicit priming-like transcriptional responses in non-primed seedlings. These findings confirmed the importance of histone marks in posttranscriptional regulation and developing the priming response to salt stress in soybean (Yung et al., 2022). Salt stress changed the expression of genes and epigenetic repressive mark, histone H3 lysine 27 trimethylation (H3K27me3) deposition in soybean (**Figure 9**). Whole-genome ChIP-seq study has identified that the inactivation of genes under salt stress strongly correlates with histone methylation, promoter or coding regions H3K27me3 methylation. Dynamic chromatin regulation through histone modifiers acts as on-and-off switches to respond to salt stress in soybean (Sun et al., 2019a). These epigenetic events control the expression of stress-responsive genes, all of which could regulate transgenerational salt stress response memory in soybean.

## Role of miRNA in soybean salt tolerance

microRNA genes are involved in various stress responses in soybean. Gma-miR169c-nuclear factor Y-A (NF-YA) modules expressed in different tissues and respond to polyethylene glycol (PEG), high salt, cold stress and abscisic acid (ABA) in soybean. Gma-miR169c exerts a negative regulatory role by repressing its target genes and modifying the various physiological response and stress-responsive genes *AtCOR15A*, *AtRD29A*, *AtRD22*, and *AtGSTU25* in Arabidopsis (Yu et al., 2019). miRNA expression was also epigenetically regulated through the process of Histone deacetylation that caused miRNA or target gene repression and silencing. Recently, a small RNA seq library identified 17 miRNAs and 31 putative target genes under salt stress in soybean. The potential targets of miR482bd-5p were *HEC1* and *BAK1*, which exhibited opposite expression patterns. Histone deacetylation analysis of miR482bd-5p showed epigenetic regulation and target gene *HEC1* was up-regulated under SAHA-salt treatment in soybean (Cadavid et al., 2020a). **Table 3** shows that the miRNA-target genes modules in salt stress response were functionally validated in soybean or Arabidopsis (Sun et al., 2015; Li et al., 2016; Pan et al., 2016; Sahito et al., 2017; Yu et al., 2023).

**Table 3:** List of Soybean miRNA for salt tolerance.

miRNA	Target gene	Transgenic	Remarks	Reference
GmmiR172a	SSAC1	Soybean	Salt tolerant	Pan et al., 2016
GmmiR172c	*Glyma01g39520*	Arabidopsis	Salt tolerant	Li et al., 2016
GmmiR172c	NNC1	Soybean	Salt tolerant	Sahito et al., 2017
GmmiR169c	NF-YA	Arabidopsis	Salt tolerant	Yu et al., 2019
GmmiR482	HEC1	Soybean	Salt tolerant	Cadavid et al., 2020a
GmmiR4368b	FBX193	Soybean	Salt tolerant	Yu et al., 2023
GmmiR399	PHO2	Soybean	Salt sensitive	Sun et al., 2015

SSAC1 (salt suppressed AP2 domain-containing), (NNC1) Nodule Number Control 1, (NF-YA) Nuclear factor-Y, (FBX193) F-box gene

## Genome editing to improve soybean salt tolerance

CRISPR/Cas based genome editing is an efficient approach to improve agronomic traits, including stress tolerance in crops without fitness costs (Zhang et al., 2021). Precise gene editing has become an indispensable routine tool for soybean functional genomics studies (**Figure 7**). CRISPR/Cas systems have been extensively utilized for traits-genes relation validation in soybean, such as photosynthesis, yield, grain quality, biotic and abiotic stress tolerance (Bao et al., 2019; Do et al., 2019a). For instance, ABA -induced transcription repressors (*AITRs*) are conserved in angiosperms, regulate ABA signaling, and are localized in the nucleus. ABA treatment increased the expression of *GmAITR*, and CRISPR/Cas9 edited Cas9-free *gmaitr36* double and *gmaitr23456* quintuple mutants showed ABA sensitivity and exhibited salt tolerance at seed germination and seedling stage in lab and field experiments (Wang et al., 2021a). In soybean, CRISPR/Cas mediated knockdown of *GmSK2-8* induced salt sensitivity during nodule formation. Whereas 2-bp deletion of *Gmnsp1b-1* caused frameshift mutation and mutants plants reduced the number of produced nodules compared with WT (He et al., 2021). An efficient CRISPR/Cas9 vector system for multiplex genome editing can target multiple sites in one or multiple genes needed for editing (Ma et al., 2015). CRISPR/Cas9-based edited *GmMYB118* mutants accumulated less proline and chlorophyll contents and showed compromised salt tolerance in soybean (Du et al., 2018). Similarly, Recently, the *GmNHX5* function was validated through CRISPR/Cas9 mediated mutant in soybean. The edited plants exhibited reduced salt tolerance compared to *GmNHX5* overexpression soybean plants. Under salt stress treatment, *GmNHX5*-OE plants exhibited higher expression of salt responsive genes *GmSOS1* and *GmSKOR*, and maintained a higher Na^+^/K^+^ ratio thus improving the soybean salt stress tolerance (Sun et al., 2021a). However, *GmNHX5*-*GmSOS1*-*GmSKOR* modules need further confirmation by genetic analysis. These findings provided a potential candidate gene for developing salt-tolerant soybean germplasm. Likewise, CRISPR/Cas9 mediated editing of *GsSOS1* and *GsNSCC* was performed and the response of mutant plants was validated against salt stress. It was observed that *GsSOS1* mutant plants exhibited a changed Na^+^/K^+^ ratio compared to *GsNSCC* mutants, which did not show any significant differences in Na^+^/K^+^ ratio under salt stress treatment (Niu et al., 2020). CRISPR-Cas9 mediated edited plants for *GmNAC06* exhibited induced proline accumulation to minimize ROS adverse effects and maintained the higher Na^+^/K^+^ ratios for ions homeostasis, thus improving the salt stress tolerance (Li et al., 2021a). Although CRISPR/Cas is efficiently utilized, polyploidy and inefficiency of transformation protocol in soybean is a major hurdle in functional genomic validation through CRISPR/Cas. For CRISPR/Cas mediated genome editing, transient hairy root and stable methods have been developed, but their genome editing efficiency largely varies in different soybean cultivars (Liu et al., 2019b). Compared to rice prime base editing by CRISPR/Cas, which allows for base substitutions still not reported in soybean. Consequently, different kinds of CRISPR/Cas systems will provide help for the identification and functional characterization of genes but also provide the opportunity for crop improvement (Chen et al., 2019).

## Conclusion and future recommendations

Soybean production is severally affected in salt and alkaline stress soil. The synergetic effects of salt and alkali stress are more deleterious and have become a prime concern for soybean production. Soybean makes a series of modifications to adapt salt stress conditions, for instance, activation of membrane transporters for Na^+^/K^+^ homeostasis, Ca^2+^ influx, antioxidant defense systems, osmotic adjustment, and hormonal regulation to minimize or removal of lethal ion. Active coordination between different cellular organelles through retrograde signalling also proved helpful in coping with salt stress conditions. Developing new salt tolerant soybean varieties with stable and higher yield across unfavorable environments could provide a sustainable supply. Vast genetic diversity and high-quality soybean genome availability help identify salt stress responsive genetic modules/switches, molecular networks and QTLs. These excellent genetic modules/switches could be exploited through transgenic or breeding approaches to develop salt resilient soybean to improve growth and yield under adversity. This review not only comprehensively enriched our understanding of the effect of salt stress on soybean but also provided genetic mechanisms that play role in salt stress tolerance in soybean. Although the investigation of salt stress responses and molecular mechanisms are well defined in cereals, especially in rice and maize plants, it still has a long way to go in soybean crops. For instance, the regulation of identified molecular switches for gene expression and signalling cascade that may regulate Na^+^/K^+^ transporters and precise coordination among them remain to be elucidated. However, our current knowledge is limited to transporters that have a role in Na+/K+ homeostasis. In response to salt stress, several cation transporters change their expression; however, their biological role is still elusive in soybean. It is also unclear that salt transporters or antiporters explained here could contribute to plant agronomic performance as well. The molecular pathways regulated by GmAKT1, GmCHXs and CIPKs their cross talk with phytohormone will further help to understand the salt stress response mechanisms in soybean. Domestication process led to the loss of important salt stress-responsive genetic loci in cultivated soybean. Although a significant variation exists among cultivated and wild soybeans for the salt response. The allelic diversity and beneficial alleles of wild soybeans could be incorporated into domesticated soybeans to improve salt tolerance. The complexity of soybean genome and lacking of efficient genetic transformation methods make soybean stress tolerance understandings further complicated. Ultimately, optimizing genetic transformation protocols for in-depth study of downstream and upstream genetic and molecular networks with advanced molecular tools will help elucidate and understand soybean salt tolerance mechanisms. Epigenetic modifications of salt responsive genes through alternative splicing also contribute to the complexity of understanding stress tolerant mechanisms. Therefore, each splicing variant needs to be verified in soybean through genetic engineering. Applying the principles of speed breeding with other efficient crop breeding approaches, like genetic engineering, genotyping by sequencing and genomic selection, could accelerate the soybean improvement for salt resilience. In the future, further improvement and integration of several omics’ approaches, such as small RNA-omics, transcriptomic, proteomic, metabolomics, ion omics and genetic engineering, could warrant to advance soybean varieties with high salt tolerance under saline conditions.

## Author contributions

CF, MH and HG conceived the idea and wrote the manuscript. MH, HG, CF and YJ prepared the figures. SL, ZY, KX, WZ, YZ, FZ and YJ helped in the literature search and assistance. MH, XY, WZ and HL supervised the work and edited the manuscript. XY, YZ and HL revised the manuscript and manage the funding resources. All authors contributed to the article and approved the submitted version.
